# Estimates of Effective Number of Breeders Identify Drivers of Decline in Mid‐Atlantic Brook Trout Populations

**DOI:** 10.1111/eva.13769

**Published:** 2024-09-30

**Authors:** Zachary L. Robinson, Jason A. Coombs, Mark Hudy, Keith H. Nislow, Andrew R. Whiteley

**Affiliations:** ^1^ Wildlife Biology Program, Department of Ecosystem and Conservation Sciences, College of Forestry and Conservation University of Montana Missoula Montana USA; ^2^ Northeast Fishery Center US Fish and Wildlife Service Lamar Pennsylvania USA; ^3^ Rockingham Virginia USA; ^4^ U.S. Forest Service, Northern Research Station University of Massachusetts Amherst Massachusetts USA

**Keywords:** brook trout, genetic monitoring, habitat fragmentation, *N*_b_

## Abstract

Brook Trout (*Salvelinus fontinalis*) populations have experienced marked declines throughout their native range and are presently threatened due to isolation in small habitat fragments, land use changes, and climate change. The existence of numerous, spatially distinct populations poses substantial challenges for monitoring population status (e.g., abundance, recruitment, or occupancy). Genetic monitoring with estimates of effective number of breeders (*N*_b_) provides a potentially powerful metric to complement existing population monitoring, assessment, and prioritization. We estimated *N*_b_ for 71 Brook Trout habitat units in mid‐Atlantic region of the United States and obtained a mean *N*_b_ of 73.2 (range 6.90–493). Our modeling approach tested whether *N*_b_ estimates were sensitive to differences in habitat size, presence of non‐native salmonids, base flow index, temperature, acidic precipitation, and indices of anthropogenic disturbance. We found significant support for three of our hypotheses including the positive influences of available habitat and base flow index and negative effect of temperature. Our results are consistent with presently observed and predicted future impacts of climate change on populations of this cold‐water fish. Importantly, these findings support the use of *N*_b_ in population assessments as an index of relative population status.

## 1 Introduction

Habitat loss and fragmentation are primary contributors to elevated extinction rates in freshwater environments (Brauer and Beheregaray 2020). Freshwater streams are vulnerable to complete barriers to dispersal (i.e., dams, culverts, unsuitable habitat sections) due to their dendritic and directional nature. This characteristic of these systems often creates numerous populations that are nearly or completely demographically and genetically independent. Thus, conservation practitioners are often faced with the challenging task of monitoring and managing numerous, declining, and spatially distinct populations (Linke, Turak, and Nel 2011; Merriam, Petty, and Clingerman 2019). Genetic monitoring is an increasingly used approach for prioritization and monitoring freshwater systems and can provide valuable insights into rates of inbreeding and loss of genetic variation, connectivity among habitat fragments, and demography (Luikart et al. 2010). Recently, there have been numerous calls to better incorporate genetic data into management and species status listing criteria at the regional and global level with an emphasis placed on estimates of effective population size (COP and CBD 2022; Garner, Hoban, and Luikart 2020; Laikre 2020).

Generational effective population size (*N*_e_) is *the* fundamental evolutionary parameter and describes the genetic properties of a population such as rates of inbreeding and loss of genetic variation, the efficacy of natural selection, and can provide demographic insights into a population. As a result, *N*_e_ is widely regarded as the gold standard of genetic metrics in conservation. However, *N*_e_ is difficult to estimate in natural populations, which, among other challenges, often have long, overlapping generations (Waples, Antao, and Luikart 2014). Estimation of generational *N*_e_ for species with overlapping generations requires detailed demographic information (Jorde and Ryman 1995; Waples et al. 2013) or multiple genetic samples spaced apart by multiple generations (Waples, Do, and Chopelet 2011; Waples and Yokota 2007). Single‐sample estimators, such as the LD method (Do et al. 2014), of effective population size have clear logistical advantages for conservation practitioners but also assume discrete generations. When single‐sample estimators are applied to mixed‐age samples in populations with overlapping generations estimates of *N*_e_ are downwardly biased and often reflect a value between effective number of breeders (*N*_b_) and generational *N*_e_ (Waples, Antao, and Luikart 2014). This ambiguity can be avoided by estimating *N*_b_ using single‐cohort samples, which estimates the effective size of the parents that gave rise to a single cohort or age class. Importantly, *N*_b_ estimates can be readily converted to *N*_e_ using a few life‐history traits (Waples et al. 2013) permitting such estimates to be placed in the context of international conservation frameworks (e.g., COP and CBD 2022).

The effective number of breeders has substantial potential for use in genetic monitoring programs. *N*_b_ can be estimated with single‐cohort genetic samples using existing, well‐tested software such as the linkage disequilibrium estimator (Do et al. 2014). Using reasonable sampling effort, this estimator is most precise for populations with small to moderate effective sizes (e.g., *N*_b_ < 500), and is therefore well‐suited for monitoring small populations of conservation concern (Do et al. 2014). The evolutionary significance of *N*_b_ is well‐established through its relationship with generational *N*_e_ (Waples 2002a; Waples et al. 2013). However, this metric also provides information relevant to both demographic and ecological processes in a way that helps discern population status. Empirical studies have illuminated *N*_b_'s relationship with factors that affect the formation of a cohort such as the number of reproductive adults (Yates, Bernos, and Fraser 2017), and early‐rearing habitat quantity and quality in organisms with localized reproduction (Whiteley et al. 2015). There has been sustained interest in using effective size estimates to make demographic inference (Luikart et al. 2021; Waples 2002b). Yet, there are mixed results for stable relationships between *N*_c_ and *N*_b_ in natural populations, which is often expressed as temporal variation in the *N*_b_/*N*_c_ ratio (Bernos and Fraser 2016; Duong et al. 2013). For use in monitoring, it is important to more clearly establish interpretive frameworks that account for evolutionary, demographic, and ecological drivers of variation in *N*_b_ within and among populations, and under what conditions *N*_b_ is suitable for genetic monitoring and assessment.

Brook Trout (*Salvelinus fontinalis*) populations are well‐suited for examination of environmental drivers of *N*_b_. Well‐studied Brook Trout populations have demonstrated relationships between *N*_b_ and stream flow during reproduction (Whiteley et al. 2015) and a relationship between *N*_b_ and adult census size (Ruzzante et al. 2016; Yates, Bernos, and Fraser 2017). Additionally, Brook Trout populations in the mid‐Atlantic region of the United States are an example of a widely distributed freshwater fish species that is declining due to habitat loss, fragmentation, and vulnerability to climate change (Hudy et al. 2008; Merriam, Petty, and Clingerman 2019). This species occupies thousands of habitat patches in the mid‐Atlantic region that are often small (<2000 ha drainage area) and are expected to have little or no population connectivity to adjacent populations (EBTJV 2016). Importantly for estimating *N*_b_, these relatively small and isolated populations minimize bias that is associated with violated assumptions such as continuous population structure or migration (Neel et al. 2013; Waples and England 2011). Brook Trout populations, along with other salmonids, are also strongly affected by access to quality spawning and early‐rearing habitat (Beard and Carline 1991; Blum, Kanno, and Letcher 2018; Petty, Lamothe, and Mazik 2005), which highlights how both abundance of mature adults and ecological factors (e.g., reproductive habitat) might influence *N*_b_ estimates. Further, the high sensitivity of Brook Trout populations to early‐life vital rates (Kanno et al. 2016) may provide a more direct link between *N*_b_ and population viability as compared to other taxa.

In this paper, we first use established theoretical work on *N*_b_ to illustrate the potential ecological insights provided by this genetic metric. This context provides a useful framework to form hypotheses regarding how habitat characteristics may influence the amount and variation in reproductive success among Brook Trout populations and ultimately influence *N*_b_ estimates. Next, we evaluated the use of *N*_b_ as a genetic monitoring metric by using empirical estimates and determining if it is influenced by factors known to affect Brook Trout status, abundance, and occupancy. We reason that because *N*_b_ reflects annual processes related to reproduction and early juvenile survival, and since recruitment dynamics are a key aspect of Brook Trout demographic patterns (Kanno et al. 2016), that *N*_b_ will provide an index of relative population status. It follows that we predict *N*_b_, along with its two primary subcomponents (number of breeders and variance in reproductive contribution) will be influenced by major drivers of population status at the basin scale. We build models of *N*_b_ that aim to identify relationships with physical, ecological, and chemical aspects of the habitat that are known to affect Brook Trout population status, abundance, or occupancy. Our results will serve broadly to advance the use of *N*_b_ in conservation monitoring and will inform its continued use for headwater salmonid conservation.

## 2 Methods

### 2.1 Relevant Theory for Genetic Monitoring With *N*_b_

Placing the theoretical description of *N*_b_ in an ecological context aids the interpretation of *N*_b_ estimates and sets expectations of its usefulness within a broader genetic monitoring program. Waples and Waples (2011) demonstrated that *N*_b_ is determined by the number of contributing breeders and the mean and variance of their reproductive contribution (Waples and Waples 2011):

(1) $$N_{b}=\frac{2S-1\;}{\frac{\sum {k_{i}}^{2}}{2S}-1}$$

where the vector of *k*_*i*_ values is the number of progeny of each parent and the number of progeny (*S*) is equal to $\frac{\sum k_{i}}{2}$, given each progeny has two parents. The potential for demographic inference based on *N*_b_ estimates is apparent from the equation above, that is, if mean and variance in progeny contribution (i.e., reproductive success) and the contributing proportion of the adult population are constant, *N*_b_ will increase proportionally with *N*_c_ (i.e., there will be a stable *N*_b_/*N*_c_ ratio). However, temporally variable *N*_b_/*N*_c_ ratios (e.g., Yates, Bernos, and Fraser 2017) illustrate that *N*_b_ is not directly dependent on the census size of mature adults in a population, but rather the number of individuals that reproductively contribute and the mean and variance of their contribution to a cohort (Waples and Waples 2011). Therefore, biological or ecological constraints on the number of contributing breeders can be reflected in empirical estimates of *N*_b_.

Many populations of conservation concern are affected by breeding site quality or quantity (Geist and Dauble 1998; Aitken and Martin 2012; Mottl et al. 2020). For example, many stream‐dwelling fish species have point distributions of reproduction (Whiteley et al. 2014a) with a finite number of suitable breeding locations (Beard and Carline 1991). In such cases, the availability of suitable reproductive habitat effectively limits the potential number of contributing breeders of one or both sexes, which can create a nonlinear temporal relationship between *N*_b_ and *N*_c_. For example, *N*_b_ can be estimated for each sex and the *N*_b_ of the population can be calculated as follows (Wright 1938):

(2) $$\frac{1}{N_{b}}=\frac{1}{4N_{\mathrm{bm}}}+\frac{1}{4N_{\mathrm{bf}}}$$

Let's assume that *N*_b_ of females (*N*_bf_) is constrained by available breeding sites and that *N*_bf_ is equal to the number of available breeding sites (i.e., females are ideal individuals and there is one female per breeding site). Ecological constraints such as this will place a limit on the maximum population value of *N*_b_ (Modified from Waples and Antao 2014):

(3) $$\lim_{N_{\mathrm{bm}}\to \infty }\;\frac{1}{4N_{\mathrm{bm}}}+\frac{1}{4N_{\mathrm{bf}}}=\frac{1}{4N_{\mathrm{bf}}}$$

In this hypothetical case of extreme breeding site limitation, *N*_b_ of the population will not exceed 4*N*_bf_, which would correspond to four times the number of breeding sites, even as the effective number of male breeders (*N*_bm_) becomes arbitrarily large. This example, albeit simplistic, further illustrates that *N*_b_ can become decoupled from the census size of mature adults for certain taxa and ecological contexts. Therefore, when *N*_b_ is employed as a genetic monitoring metric, it is often simultaneously providing genetic, demographic, and ecological insights into a population, which may strongly correlate with adult abundance, reproductive habitat quantity or quality, or early‐life mortality depending on the ecological conditions experienced by the progenitors of a cohort or the progeny themselves (Bacles et al. 2018; Whiteley et al. 2015). We, therefore, predict that, when used as a genetic monitoring metric, *N*_b_ will provide information about cohort‐specific ecological and demographic processes related to reproductive contribution and factors that limit it.

### 2.2 Brook Trout Sampling

**FIGURE 1:**
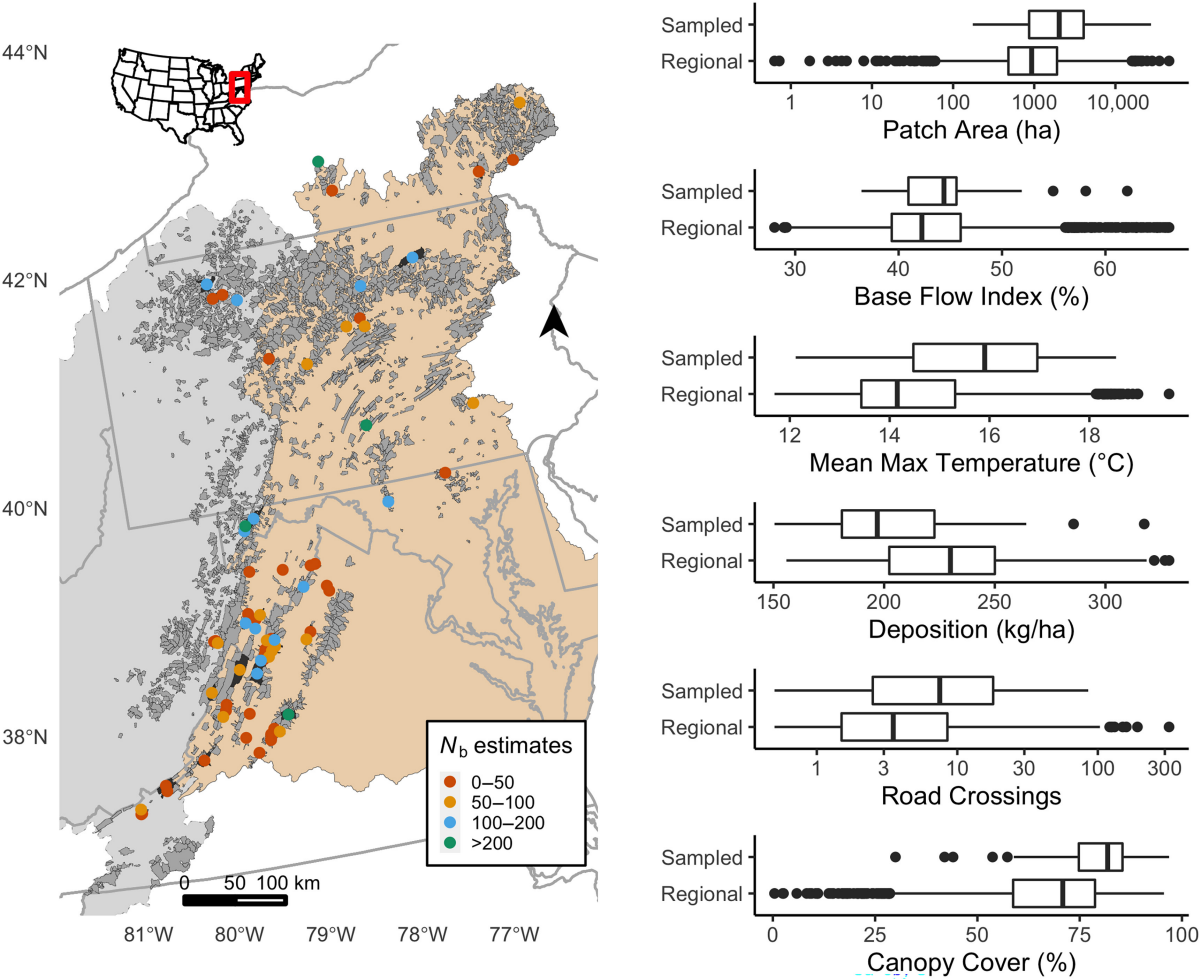
Map of study area in the mid‐Atlantic region of the United States. The brown and gray shaded areas indicate the Chesapeake Bay and Ohio River region, respectively. Each point on the map corresponds to a sampled EBTJV Brook Trout habitat patch (*n* = 71) colored with respect to the magnitude of ${\hat{N}}_{b}$. The numerous dark gray drainage areas (*n* = 2773) represent unsampled habitat patches. Box plots present the distribution of the variables used in modeling for the sampled habitat patches (Sampled) compared to all habitat patches in the focal region (Regional).

A consortium of state and federal agencies collected 8121 tissue samples from 71 Brook Trout habitat patches in the mid‐Atlantic region of the United States from 2009 to 2018 (Figure 1). A habitat patch is defined as an area of contiguous catchments (seventh level, 14‐digit hydrologic unit codes; USGS 2012) within which fluvial habitats are continuously occupied by Brook Trout (EBTJV 2016). Of the sampled habitat patches, 59 occur in the Chesapeake Bay basin, and 12 occur in eastern Ohio River basin. Biologists and managers within each jurisdiction played a major role in selecting habitat patches of interest for genetic monitoring. We chose habitat patches as the unit of sampling because, in this region, they often represent a discrete, biological population for which genetic parameters can be accurately estimated (Whiteley et al. 2014b), and also corresponds to the scale of management action. Importantly, single‐cohort samples of Brook Trout can be obtained because age‐0 Brook Trout can be easily distinguished based upon length‐frequency histograms during their first summer (Hudy et al. 2000). Upon capture, total length was measured and a small (<1 cm^2^) caudal fin clip obtained for genetic analysis and the fish was immediately returned to the approximate point of capture. Brook Trout were captured using one‐pass electrofishing using the sampling protocol from Whiteley et al. (2012), which has been shown to produce unbiased estimates and avoid family overrepresentation. Briefly, juvenile (age‐0) Brook Trout were sampled at three equidistant locations within the habitat patch with a target of 25 individuals from each location. In certain cases, sample sizes less than recommended (*n* < 75) were included for analysis (minimum *n* = 17), because it is not unusual for habitat patches in this region to have age‐0 abundance less than 50 (e.g., Robinson et al. 2017). Accurate estimates of *N*_b_ can be obtained with samples less than 75, particularly when true *N*_b_ is less than the sample size (Ackerman et al. 2017; Waples 2006), which is likely in these populations.

### 2.3 Genotyping

DNA was extracted from all age‐0 Brook Trout tissue samples and genotyped using two genotyping methods. Samples collected prior to 2015 (5397 DNA samples from 45 habitat patches) were genotyped at 8‐microsatellite loci (*SfoC113*, *SfoD75*, *SfoC88*, *SfoD100*, *SfoC115*, *SfoC129*, *SfoC24*; King et al. 2012) and *SsaD237* (King, Eackles, and Letcher 2005). These microsatellite markers have been extensively tested and have not exhibited systematic deviations from HW proportions or LD in mixed‐aged samples from hundreds of Brook Trout populations (e.g., Annett et al. 2012; Kanno, Vokoun, and Letcher 2011; Kazyak et al. 2022; Robinson et al. 2017). Samples collected from 2016 on (2754 DNA samples from 43 habitat patches) were genotyped using genotyping‐in‐the‐thousands by sequencing (GT‐seq; Campbell, Harmon, and Narum 2015). The GT‐seq marker panel was developed by Idaho Department of Fish and Game and included 240 amplicons, each possessing a single nucleotide polymorphism (SNP) targeted for genotyping (M. Campbell, *personal communication*). Sequencing libraries were prepared using the protocol described in Campbell, Harmon, and Narum (2015) and sequenced on an Illumina NextSeq 500 instrument. We tested the 240 GT‐seq loci for deviations from HW proportions and for LD in program GENEPOP version 4.7.5 (Rouseset 2008), after which 167 polymorphic SNP‐loci were retained that genotyped in at least 50% of the samples (Appendix S1). We required that samples successfully genotype at 75% of loci to be retained in further analyses.

### 2.4 Genetic Analysis

For each habitat patch sampled, we estimated genetic variation and *N*_b_. To describe genetic variation, we calculated observed and expected heterozygosity and *F*_IS_ using the statistical computing software *R* v4.0.3 (R Core Team 2020) and the *R* package ‘hierfstat’ (Goudet 2014). Effective number of breeders was estimated for each habitat patch using the LD method in NeEstimator v2.1 (Do et al. 2014). We estimated *N*_b_ assuming a monogamous mating system, based upon the observation of that 80% of reproductive individuals contribute to a single family in two Brook Trout populations (Coombs 2010). Importantly for this investigation, the mating system assumption will influence the absolute value of *N*_b_, but not the relative value among habitat patches (Waples 2006). *N*_b_ estimates were derived using a minimum allele frequency cutoff (*P*_crit_) of 0.02, which has been shown to provide an adequate balance between precision and bias across sample sizes (Waples and Do 2008). We report uncertainty in our *N*_b_ estimates with 95% confidence intervals produced using the jackknife method across individuals within each sample.

Waples, Antao, and Luikart (2014) demonstrated bias in genetic estimates of *N*_b_ in population with overlapping generations and age structure such as Brook Trout. Additionally, they provide a bias correction that can be applied to empirical estimates. This bias correction requires the true *N*_b_/*N*_e_ ratio, which can be calculated with a life table in the program AgeNe (Waples, Do, and Chopelet 2011). We chose not to apply this bias correction for two reasons. First, although a life table is available from an intensively studied Brook Trout population in Massachusetts, USA (Letcher et al. 2014), the correction will have no impact on relative *N*_b_ estimates among patches without estimated vital rates for each habitat patch. Secondly, the estimated bias correction for the same intensively studied Massachusetts population referenced above was minimal at 3.4% (Whiteley et al. 2015), and is likely insignificant compared to the potential bias generated by sampling (e.g., Whiteley et al. 2012).

*N*_b_ estimates based upon the two different marker types are not expected to be significantly biased relative to one another and can be directly compared (Waples and Do 2010). However, we do expect higher precision in estimates generated by the GT‐seq marker panel due to the higher number of loci (Luikart et al. 2021). An empirical comparison of the marker types used in this dataset supports this expectation (Figure S1).

Sibship reconstruction can provide context to *N*_b_ estimates through inferring the number of families and the variance of family sizes within a sample. We used Colony2 v2.0.6.5 for sibship reconstruction (Jones and Wang 2010). Colony was run using the full‐likelihood and pairwise likelihood combined method specifying medium precision and medium run length. Each run was conducted without updating or specifying allele frequencies and assuming no inbreeding. We assumed a polygamous mating system for sibship reconstruction because, as previously stated, we expect some degree of polygyny in Brook Trout populations. We specified a genotyping error rate of 0.005 for both genetic marker panels. For each habitat patch sample, we summarized the total number of full‐sibling families and variance in full‐sibling family size. Variance in full‐sibling family size was estimated using a fitted negative binomial distribution using the *R* package *fitdistrplus* (Delignette‐Muller and Dutang 2015), because reproductive success in salmonids is commonly over‐dispersed (Koch and Narum 2021). Although half‐sibling relationships would better reflect the reproductive success of individual parents (e.g., Equation 1), they are difficult to accurately infer using sibship reconstruction without parentage when using modest genetic marker panels (Flanagan and Jones 2019). As a result, we used the number of full‐sibling families and variance in full‐sibling family size as a proxy for the number of successful breeders and their variance of reproductive success.

The program Colony also provides a sibship‐based estimate of *N*_b_ (Wang 2009), and we report these for comparison to the LD‐based estimates. We preferentially used *N*_b_ estimates from the LD method for subsequent modeling because it was reasonable to assume that some of the population sample sizes were less than true *N*_b_. The LD method has an adjustment that minimizes bias when sample size is less than true *N*_b_ (Waples 2006, 2024), whereas the sibship‐based estimate is often biased low (Ackerman et al. 2017). The Colony‐derived *N*_b_ estimates were made using a separate execution of Colony assuming a monogamous mating system with all other settings unchanged. An assumption of a monogamous mating system was found to produce the most accurate sibship‐based *N*_b_ estimates in another salmonid species that exhibited approximately 80% monogamy (Ackerman et al. 2017), and is consistent with the assumptions used for the LD‐method.

### 2.5 Variable Selection

We hypothesized that *N*_b_ will be useful in inferring the population status within Brook Trout habitat patches based upon previous research in this species. We test this hypothesis, albeit indirectly, by determining if *N*_b_ is related to the factors that are known to affect occupancy, abundance, and status of Brook Trout populations. We include variables that are intended to represent habitat size, stream flow, temperature, competition with non‐native salmonids, an index acidic precipitation, and anthropogenic disturbance (Table S1). For habitat size, we calculate habitat patch size as the product of NHDplus V2 stream length (km) and habitat patch area (ha) (EBTJV 2016) divided by 1000 following Whiteley et al. (2012).

We included base flow index and mean max temperature due to their direct relationships with rising stream temperature and the vulnerability of this Brook Trout to climate change (Bassar et al. 2016; Trumbo et al. 2014). Base flow index (BFI) was obtained from the U.S. Geological Survey (Wieczorek 2018) and we used the mean value within a habitat patch. Within a habitat patch, BFI is intended to reflect relative groundwater contribution to stream flow, which has an empirically observed, positive effect on Brook Trout reproduction and stability of stream flow and temperature (Curry and Noakes 1995; Nuhfer, Zorn, and Wills 2017). Brook Trout populations have been negatively affected by rising stream temperatures and are expected to decline further with climate change (Bassar et al. 2016). Within the mid‐Atlantic region, we predict the range of stream temperatures experienced would produce a negative, approximately linear relationship between *N*_b_ and stream temperature. We used mean maximum annual air temperature from 1991 to 2020 from the PRISM climate group as an index of stream temperature.

Habitat degradation due to human activities has negatively impacted Brook Trout throughout their range including species introductions, acid deposition from industrial activity, and human development (Hudy et al. 2008; Merriam, Petty, and Clingerman 2019). The presence or absence of non‐native salmonids was obtained from the Eastern Brook Trout Joint Venture habitat patch layer (EBTJV 2016) and field observations during sampling. This metric was used to account for negative effects of competition with non‐native salmonids within the habitat patch (Hitt, Snook, and Massie 2017). Acidic precipitation and low stream pH has had a significant negative effect on Brook Trout populations (Hudy, Downey, and Bowman 2000; Nislow and Lowe 2003). Hence, we used the within‐patch average sum of total nitrogen and sulfur deposition, hereafter acid deposition, using data from 2000 to 2002 and 2010–2020 as an index of acidifying precipitation (NADP 2022). We included the number of road‐stream crossings per hectare and percent canopy cover from the 2011 National Land Cover Database as a proxy for human disturbance and land use, which is negatively associated with Brook Trout occupancy (Merriam, Petty, and Clingerman 2019). All spatial datasets were summarized using the geographic information software QGIS (QGIS Development Team 2020). All variables excluding the presence or absence of non‐native salmonids were *z*‐score standardized prior to modeling.

### 2.6 Statistical Analysis

${\hat{N}}_{b}$ was modeled with a Bayesian mixed effect, generalized linear model with a Gaussian error distribution and log link function using weighted observations in the statistical program JAGS version 4.3.0 (Plummer 2003). We constructed and fit the model in the statistical computing program *R* and called JAGS using the package ‘R2jags’ (Su and Masanao 2015). Slope coefficients of all seven predictor variables and a random intercept were estimated using uninformative, normally distributed priors centered on zero. The model was fit with a random intercept term based on the US state that contained the habitat patch. We selected these political boundaries for the random intercept because sampling and site selection was most often conducted by different entities in different states. Additionally, state boundaries roughly correspond to geographical clusters of environmentally similar Brook Trout populations (Zhang et al. 2008). Predictor variables with missing values were included and values were drawn from a fitted normal distribution corresponding to each variable (Appendix S2).

For inclusion in the model, we required that each sample generate a positive, finite point estimate of *N*_b_. Additionally, 26 of the 71 habitat patches had multiple cohorts sampled (i.e., years) and we used a single estimate for modeling purposes generated by the harmonic mean of cohort‐specific *N*_b_ estimates. To accommodate differing levels of uncertainty, our *N*_b_ estimates were given relative weights in the model based on the width of the mean standardized, 95% jack‐knifed confidence interval width corresponding to each *N*_b_ estimate. If the upper bound confidence interval included infinity, the observation was given a weight based on two times the largest finite confidence interval within the dataset. In cases where we used the harmonic mean of multiple *N*_b_ estimates for a habitat patch, we used the arithmetic mean of the confidence interval widths for weighting. These relative weights were intended to account for differences in precision generated by field sampling and the two different marker panels used.

The number of full‐sibling families ($N_{\mathrm{FAM}}$) and the variance in full‐sibling family size (${\sigma^{2}}_{\mathrm{FS}}$) were modeled using the same seven predictor variables and similar model formulation as ${\hat{N}}_{b}$. These two models of sibship‐based summary statistics differed only in that we did not weight observations. Although the number of sampled individuals could provide a proxy for uncertainty, the primary source of uncertainty among the number of observed families is sampling effort or probability of detection. Weighting by sample size could produce spurious results because of its relationship to the quantities of interest, that is, few samples could occur due to few individuals being present or due to less sampling effort or efficiency. Unfortunately, total sampling effort was not recorded consistently among collections and is not available for use. In cases where we had multiple cohort collections for a habitat patch, we generated a single value of each sibship‐based summary statistic using a sample size weighted mean.

Where applicable, we express statistical significance of effects using the probability of direction (*pd* ≥ 0.95) (Makowski et al. 2019), which is similar to determining whether the 90% credible intervals include a zero‐effect size. We fit the model with five chains of 10,000 adaptive phase iterations and 50,000 estimation iterations with a thin rate of 2. Model convergence was evaluated by visually inspecting chains with the *R* package ‘mcmcplots’ (Curtis 2015) and the potential scale reduction factor (diagnostic values <1.1 indicate good chain mixing; Gelman and Rubin 1992). Goodness of fit of the models was accessed by posterior predictive checks using Bayesian *p*‐value (Gelman 2013) and visual evaluation of residuals and predicted values.

### 2.7 Relationship of ${\hat{N}}_{b}$ With Existing Brook Trout Population Assessments

Concordance between independent Brook Trout population assessments and *N*_b_ estimates would provide additional support that *N*_b_ performs well as a genetic monitoring metric and is potentially linked to population persistence and occupancy in mid‐Atlantic Brook Trout populations. For this exploratory analysis, we used a composite habitat integrity score and future security score generated by the organization Trout Unlimited (TU) (Fesenmyer et al. 2017). Habitat Integrity is a percentile scaled composite score that represents aforementioned factors known to influence Brook Trout populations including anthropogenic land use (e.g., Riparian forest cover, percent agriculture, stream‐road crossings) and acid deposition. The future security score used by TU is a percentile scaled estimate of stream temperature. Both future security and habitat integrity scores were estimated using TU habitat units and the average value was taken when multiple units were within an EBTJV habitat patch. We also use predicted probability of occupancy from U.S. Geological Survey's Spatial Hydro‐Ecological Data Systems (SHEDS) as a predictor of ${\hat{N}}_{b}$ (Walker, Letcher, and Hocking 2021). Predicted occupancy was estimated at the catchment scale and the average value was taken from catchments within an EBTJV habitat patch.

We constructed three models using habitat integrity, future security, and probability of occupancy each as a single predictor variable of ${\hat{N}}_{b}$. All three variables have values that range from zero to one and were not transformed prior to modeling. We used a Bayesian mixed effect, generalized linear model with a Gaussian error distribution and log link function using weighted observations in the statistical program JAGS. Consistent with the models above we used US state as a random intercept and used the width of jack‐knifed confidence intervals of ${\hat{N}}_{b}$ to weight observations. We fit the model with five chains of 1000 adaptive phase iterations and 15,000 estimation iterations with a thin rate of 1. We evaluated model convergence using potential scale reduction factor and goodness of fit using Bayesian *p*‐value.

## 3 Results

### 3.1 Genetic Summary

All 8121 Brook Trout tissue samples from 71 habitat patches were extracted and genotyped with a minimum of 75% genotyping success. Median sample size was 67.5 (mean 76.6, range 17.0–510). As expected, estimates of mean population heterozygosity were consistently lower when estimated with the biallelic SNP markers compared to the microsatellite marker panel (Table S2). Forty‐five of the habitat patches had at least one microsatellite‐based estimate of expected heterozygosity with an average of 0.676 (range 0.376–0.796). Forty‐three of the habitat patches had at least one estimate of biallelic, SNP‐based mean expected heterozygosity with an average of 0.196 (range 0.064–0.311). Estimates of heterozygosity for both marker types were available for 17 habitat patches from different cohorts and these estimates exhibited a Pearson's *r* of 0.317 (*t* = 1.29, df = 15, *p* = 0.215). Mean population *F*_IS_ was 0.009 (range −0.128 to 0.134) for microsatellites‐based estimates and 0.0260 (range −0.041 to 0.081) for SNP‐based estimates.

We obtained at least one positive and finite point estimate of *N*_b_ for all 71 habitat patches using the LD‐method. Of the 106 population‐cohort samples, we removed one estimate that produced a negative point estimate and retained four estimates that had infinite upper confidence intervals (Table S2). Median ${\hat{N}}_{b}$ was 46.8 (mean 74.9, range 6.9–493.1) including habitat patches for which the harmonic mean of repeated samples was used (Figure 1). In general, repeated estimates of ${\hat{N}}_{b}$ were similar among cohorts. Excluding one habitat patch, the maximum difference among ${\hat{N}}_{b}$ estimates for a single habitat patch with multiple sampled cohorts was 45.4 on average (median 31.75, range 1.0–210.8). However, a habitat patch in Maryland, had an estimate of ${\hat{N}}_{b}=1811.4$ (95% CI 308–∞) based on a sample size of 43, compared to the cohort 2 years prior which had an ${\hat{N}}_{b}$ = 285.2 (95% CI 134–2715) with a sample size of 75 (Table S2). After taking the harmonic mean of ${\hat{N}}_{b}$ for modeling purposes, we retained an ${\hat{N}}_{b}$ = 493.1 for this habitat patch, which lies within the confidence intervals of both estimates and within a biologically plausible range.

Positive and finite point estimates of *N*_b_ using the sibship‐based method were also obtained for all 71 habitat patches, and all 106 population‐cohort estimates had finite point estimates and confidence intervals (Table S2). Median ${\hat{N}}_{b}$ was 41.0 (mean 56.0; range 4.0–169.0) including habitat patches for which the harmonic mean of repeated samples was used. Considering all population‐cohort samples that produced a finite point estimates and confidence intervals using both estimation methods (*n* = 101), sibship‐based estimates were significantly correlated with LD‐based estimates (Pearson's *r* = 0.836, *t* = 15.16, df = 99, *p* < 0.001). However, the LD‐based estimates were greater than sibship‐based estimates by 20.6, on average (median 9.40). Additionally, the sibship‐based estimates were significantly correlated with sample size (Pearson's *r* = 0.307, *t* = 3.21, df = 99, *p* = 0.002), whereas the LD‐based estimates were not (Pearson's *r* = 0.122, *t* = 1.22, df = 99, *p* = 0.225). These results support our prediction that sibship‐based estimates may be biased low due to sample size, and as a result, the LD‐based estimates were used for all subsequent statistical modeling.

### 3.2 Statistical Analysis

Collinearity among independent variables was minimal and the results of the generalized linear model indicate that model performance was adequate to address our hypotheses. The mean, absolute value of all pairwise correlations (Pearson's |*r|*) among the independent variables was 0.180 (range 0.026–0.490). The maximum correlation among variables (*r* = 0.490) occurred between deposition and BFI (Table S3). The distribution of variables in our sample was, in general, representative of the broader mid‐Atlantic region, although we sampled warmer streams with higher percent canopy cover on average (Figure 1). In terms of model performance, there was no clear pattern between model residuals and predicted values. Model residuals were approximately normally distributed for models of ${\hat{N}}_{b}$ and $N_{\mathrm{FAM}}$ but right skewed for ${\sigma^{2}}_{\mathrm{FS}}$ (Figure S2). Adequate model performance for models of ${\hat{N}}_{b}$ was further supported by our Bayesian *p*‐value of 0.629, suggesting modest upward bias of predicted values. However, we observed consistent upward bias of predicted values for $N_{\mathrm{FAM}}$ and ${\sigma^{2}}_{\mathrm{FS}}$, with Bayesian *p*‐values of 0.995 and 1.00, respectively. We observed sufficient model convergence with a maximum potential scale reduction factor of 1.03 considering all estimated parameters.

**FIGURE 2:**
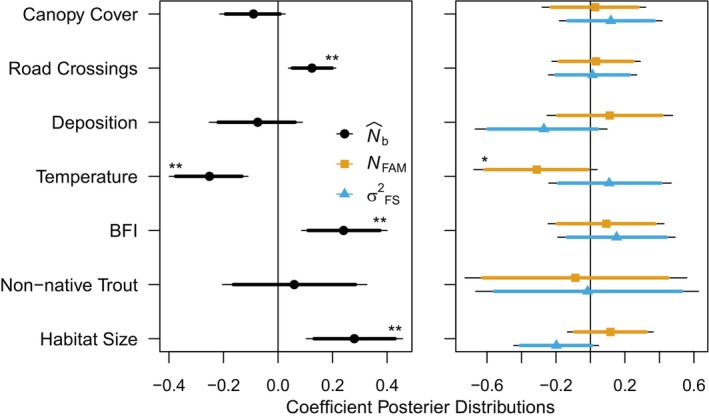
Posterior distribution of slope coefficients for models of ${\hat{N}}_{b}$, number full‐sibling families (*N*_FAM_), and variance in family size (${\sigma^{2}}_{\mathrm{FS}}$). The mean estimate (point), 90% (darker line) and 95% (lighter line) credible intervals are reported. One or two asterisks denote when the 90% and 95% credible intervals do not overlap zero, respectively.

Our generalized linear mixed model supported the hypotheses that quantity of habitat, temperature extremes, and the relative contribution of groundwater to surface flow influenced ${\hat{N}}_{b}$ within a habitat patch (Table 1). We found that habitat patch size had a significant, positive relationship with ${\hat{N}}_{b}$, and the direction of effects on $N_{\mathrm{FAM}}$ (positive) and ${\sigma^{2}}_{\mathrm{FS}}$ (negative) are both concordant with the estimated effect on ${\hat{N}}_{b}$. Base flow index also had a significant positive relationship with ${\hat{N}}_{b}$ and positive direction of effect on $N_{\mathrm{FAM}}$ and ${\sigma^{2}}_{\mathrm{FS}}$. Mean maximum annual temperature had a significant, negative relationship with ${\hat{N}}_{b}$ and the direction of effects on $N_{\mathrm{FAM}}$ (negative) and ${\sigma^{2}}_{\mathrm{FS}}$ (positive) are both concordant with the estimated effect on ${\hat{N}}_{b}$ (Figure 2). We found little support for effects of acid precipitation deposition and non‐native salmonid presence on ${\hat{N}}_{b}$.

**TABLE 1:** Mean parameter estimates and probability of direction (*pd*) for models of ${\hat{N}}_{b}$, number full‐sibling families (*N*_FAM_), and variance in family size (σ_FS_).

Coefficient	${\hat{N}}_{b}$		$N_{\mathrm{FAM}}$		${\sigma^{2}}_{\mathrm{FS}}$	
	Mean	*pd*	Mean	*pd*	Mean	*pd*
Habitat size	0.280	0.999	0.116	0.820	−0.197	0.943
Non‐native trout	0.059	0.666	−0.087	0.605	−0.017	0.522
Base Flow Index	0.240	0.999	0.092	0.707	0.152	0.809
Temperature	−0.252	>0.999	−0.311	0.958	0.109	0.722
Deposition	−0.074	0.808	0.112	0.729	−0.270	0.923
Road crossing	0.124	0.997	0.033	0.598	0.012	0.541
Canopy cover	−0.090	0.935	0.028	0.577	0.118	0.782
Mean intercept	3.513	—	3.466	—	0.931	—

**FIGURE 3:**
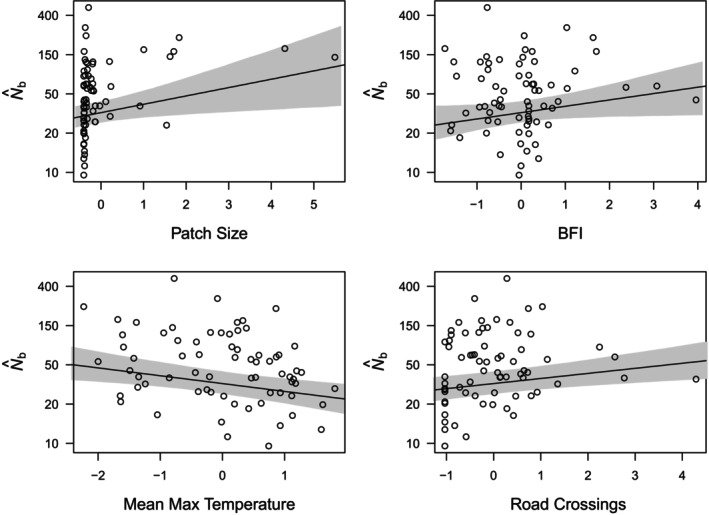
Modelled linear relationships between ${\hat{N}}_{b}$ and the four most supported variables. The gray area represents 95% credible intervals of the slope coefficient. The relationships are presented using the mean intercept, and with back‐transformed *N*_b_ estimates.

Contrary to our predictions, variables meant to reflect human habitat disturbance had positive effect size estimates on ${\hat{N}}_{b}$. Road crossings per hectare had a significantly positive relationship with ${\hat{N}}_{b}$ and percent canopy cover had a negative relationship with *N*_b_ (Figure 3). However, their effects on $N_{\mathrm{FAM}}$ and ${\sigma^{2}}_{\mathrm{FS}}$ were ambiguous (Figure 2). Notably, Bayesian indicator variable selection (Kuo and Mallick 1998) provided further support for the inclusion of habitat size, base flow index, mean max temperature, and road crossing per hectare in the model of ${\hat{N}}_{b}$ (Appendix S3).

### 3.3 Relationships With Existing Brook Trout Population Assessments

*N*_b_ estimates were significantly positively related to two out of the three independent metrics of relative population status. The mean posterior slope coefficient of the TU future security score was 0.648 (CrI 0.260–1.044). The mean posterior slope coefficient of the SHEDS probability of occupancy was 0.880 (CrI 0.478–1.38). Finally, the mean posterior slope coefficient of the TU habitat integrity score was −0.458 (CrI −1.18 to 0.284). Bayesian *p*‐values indicated adequate goodness of fit for these models with 0.530, 0.616, and 0.476 for future security score, probability of occupancy, and habitat integrity score, respectively. We observed adequate model convergence as indicated by visual inspection of chains and by the maximum potential scale reduction factor (< 1.01).

## 4 Discussion

Our results demonstrate a relationship between known drivers of population status and single‐sample estimates of effective number of breeders in a set of fragmented Brook Trout populations. Smaller estimates of *N*_b_ were associated with less available habitat, higher temperatures, and less influence of groundwater on surface flow. These relationships emphasize the importance of conservation activities that increase the amount of interconnected Brook Trout habitat (Wood, Welsh, and Todd Petty 2018), mitigate increasing stream temperatures due to climate change, and maintain or improve hyporheic exchange (Weber et al. 2017). Our results demonstrate that *N*_b_ is sensitive to factors of high relevance to conservation practitioners, is in concordance with independent population assessments, and supports that there is value added to genetic monitoring programs by incorporating *N*_b_ estimates.

Traditional genetic monitoring using estimates of genetic variation (e.g., heterozygosity, allelic richness) and genetic structure (e.g., *F*_ST_ among populations) provide insights into the past because these factors will be influenced by *N*_e_, gene flow, and population bottlenecks on the temporal scale of 10's to 100's of generations. In contrast, as we illustrate in Section 2.1, *N*_b_ is determined by the number of successfully reproducing parents, the environmental conditions under which those parents reproduced, and the conditions affecting early rearing success prior to sampling the focal cohort. The importance of reproductive habitat, juvenile abundance, and survival has been well‐demonstrated in Brook Trout populations and is particularly important in small habitat fragments (Bassar et al. 2016; Kanno et al. 2016; Letcher et al. 2007). Gaining insights into these processes have often been difficult, time‐intensive, and imprecise. For example, redd (nest) counts provide an index of reproduction but are often fraught with biological and sampling uncertainty (Dunham, Rieman, and Davis 2001). Another benefit of *N*_b_ estimation is that it can readily be converted to generational *N*_e_ with the use of a few life‐history traits (Waples et al. 2013), thus allowing estimates to be placed in the context of existing conservation genetic theory and frameworks (e.g., 50/500 rule; Franklin (1980)). Importantly, our work demonstrates that *N*_b_ estimated across populations can identify factors likely influencing reproductive processes across a broad spatial scale and represents a valuable supplement to existing population prioritization schemes.

Mid‐Atlantic Brook Trout are vulnerable to climate change and the results of our model demonstrate a link between climatic variables and a genetic metric in this cold‐water species. During the last 50 years, water temperature in the mid‐Atlantic region increased faster than air temperature at a rate of 0.028°C per year (Rice and Jastram 2015) and is negatively associated with Brook Trout occupancy (DeWeber and Wagner 2014; Merriam, Petty, and Clingerman 2019). We found a strongly negative effect of mean maximum annual air temperature on ${\hat{N}}_{b}$ in Brook Trout populations in this region. Additionally, we found that base flow index was positively associated with ${\hat{N}}_{b}$. High base flow index is associated with stability of stream flow and low sensitivity of water temperature to changes in air temperature (Trumbo et al. 2014). High base flow index may also indicate that a habitat patch has higher quality spawning habitat due an abundance of hyporheic exchange (Curry and Noakes 1995), which would contribute to increased reproductive success on average. The importance of temperature on Brook Trout is further emphasized by our exploratory analysis of two independent population assessments. The positive relationship between SHEDS occupancy model is likely driven by temperature, as it is the most significant effect in the occupancy model (Walker, Letcher, and Hocking 2021). TU's future security score represents a transformed estimate of stream temperature, and therefore, the positive association observed here is unsurprising; however, these results lend further support to the use of stream temperature as an index of future security.

Contrary to our hypotheses, our model did not support the inclusion of variables meant to represent competition with non‐native salmonids, acidification, and anthropogenic land use. Brook Trout and non‐native salmonids have been observed directly competing for spawning habitat (Grant, Vondracek, and Sorensen 2002) and non‐native salmonids have displaced or extirpated populations of Brook Trout (Kanno, Kulp, and Moore 2016; Larson and Moore 1985). Both phenomena provide an explicit mechanism through which non‐native competitors would reduce the absolute number of breeders in a Brook Trout population. We conclude that presence or absence of non‐native salmonids does not affect ${\hat{N}}_{b}$ in our collections. However, we hypothesize that a metric that better accounts for the relative abundance of non‐native salmonids would reveal the well‐documented, negative effects of competition.

Acidification has had a consistent, negative impact on Brook Trout populations throughout their range (Hudy, Downey, and Bowman 2000; Nislow and Lowe 2003). However, our index of deposition was likely inadequate to capture a biologically relevant summary of the geochemical conditions in a Brook Trout habitat patch. The population response to acidifying precipitation is complex and depends not only on the quantity of acidifying precipitation but also on buffering capacity of the catchments and aggravating factors (e.g., aluminosilicate deposits; Schofield and Trojnar 1980). Many studies have also documented negative associations with anthropogenic land use (e.g., agriculture, residential development, and deforestation) and Brook Trout occupancy at a variety of spatial scales (DeWeber and Wagner 2014; Kanno et al. 2015; Merriam, Petty, and Clingerman 2019). We found no support for a positive effect of percent canopy cover, which was used as an index of anthropogenic disturbance of forests. The difference between prior studies on Brook Trout distribution and our results may simply be an artifact of *N*_b_ estimates being conditional on occupancy and given that a patch is occupied, relatively less canopy cover does not have a marked, negative impact on Brook Trout in the mid‐Atlantic region.

Our results also revealed an unexpected pattern involving a strong positive relationship between ${\hat{N}}_{b}$ and road crossing per hectare. This result is unexpected because of the negative effects of forestry‐based roads on Brook Trout spawning habitat through the deposition of silt and other fine sediments have been well understood for over half a century (Saunders and Smith 1965), and directly affect the reproductive processes that are reflected in ${\hat{N}}_{b}$ (Hartman and Hakala 2006). However, the estimated negative relationship between ${\hat{N}}_{b}$ and percent canopy cover and TU's habitat integrity score suggests that this counter‐intuitive relationship with indices of human disturbance is relatively robust. We find two potential interpretations of these data compelling. First, the opportunistic, nonrandom sampling of habitat patches may have given rise to a spurious correlation involving road access. Second, as a popular game fish, it may be that robust Brook Trout populations are attractive for recreational development and are more likely to have more stream‐road crossings and human development within that habitat patch. A similar hypothesis was put forth in Clarke et al. (2024) suggesting that preferential human development around large, productive water bodies may explain a positive correlation found between *N*_e_ and human development in diadromous fishes.

In our models, only two variables, habitat size and temperature, had concordant direction of effects on both the number of full‐sibling families and variance of full‐sibling family size as their effect on ${\hat{N}}_{b}$ would suggest. Although both sibship‐based metrics are indices with direct effects on ${\hat{N}}_{b}$, they may lack resolution due to differing sampling effort among habitat patches in our application. Additionally, it is likely that certain ecological drivers of *N*_b_ primarily affect one demographic component of the estimate (e.g., the number of contributing breeders). For example, *N*_b_ did not increase in a Brook Trout population following removal of non‐native competitor, despite an increase in the absolute number of contributing breeders, due to an increase in variance of reproductive success (Miller, Dieterman, and Hoxmeier 2019). Nonetheless, sibship‐based summary statistics can aid in the interpretation of *N*_b_ estimates and potentially illuminate drivers of temporal or spatial variation in *N*_b_ (Whiteley et al. 2015).

The complex evolutionary, ecological, and demographic factors that influence *N*_b_ estimates can complicate their interpretation and application to conservation and management. In Section 2.1, we provide a theoretical example of how *N*_b_ can become decoupled from the absolute number of potential breeders and how environmental factors (i.e., limited spawning habitat) could strongly influence estimates. An empirical example in Atlantic salmon (*Salmo salar*) illustrates this point, lower ${\hat{N}}_{b}$ was associated with intermediate spawning aggregations where highly competitive males can dominate reproduction at a few breeding sites. Conversely, when breeding sites are diffuse or abundant, there is less variance in reproductive success and thus higher ${\hat{N}}_{b}$. Complicating matters, high‐density spawning aggregations appear to induce scramble sexual competition resulting in lower variance in reproductive success and elevate ${\hat{N}}_{b}$ (Bacles et al. 2018). These examples highlight that within and among populations variation in *N*_b_ will not always be conducive to straightforward demographic or ecological inference, such as inferring ${\hat{N}}_{c}$ from ${\hat{N}}_{b}$, due to complex density‐dependent and environmental factors (Yates, Bernos, and Fraser 2017). However, our approach of comparing *N*_b_ among populations is consistent with the observation that *N*_b_ is relatively stable within populations of Brook Trout and varies dramatically among populations, likely corresponding to reproductive habitat quantity and quality (Bernos and Fraser 2016; Whiteley et al. 2015).

### 4.1 Caveats

Our approach using nonrandom sampling and a modest sample size (*n* = 71) represents one limitation of this work. The strength of our findings is derived from their congruence with previous research on this species, including the significance of climate‐related variables and habitat quantity, and the ability to illuminate such relationships with single‐cohort genetic samples. Notably, sampling age‐0 individuals prior to recruitment may generate estimates that deviate from the evolutionarily significant value of *N*_b_ and may be more dependent on reproductive and early‐rearing habitat. The magnitude of a cohort's temporal change in *N*_b_ is expected to be dependent on how pre‐recruitment mortality reshapes the reproductive success of the progenitors of the cohort (Waples, Do, and Chopelet 2011). This work would benefit from future clarification of the magnitude of intra‐cohort temporal change in *N*_b_ and more generally *N*_b_’s relationship to near‐term persistence probability in this and other species.

Sampling with the intent to estimate *N*_b_ is difficult in stream‐dwelling Brook Trout populations due to continuous population structure and the inherent vulnerability of stream systems to fragmentation. Many mid‐Atlantic Brook Trout populations exist in small habitats that can be described as discrete populations that generate unbiased estimates of the population parameter *N*_b_ (Whiteley et al. 2012); however, larger populations or metapopulations exist in this region (e.g., Huntsman et al. 2016). Even without adding the complexity of metapopulation dynamics or barriers to movement, larger habitat patches have continuous genetic structure that is best described with the concept of genetic neighborhoods (Wright 1946), rather than as single, panmictic populations. Pooling individuals from multiple genetic neighborhoods creates a two‐locus Wahlund effect or mixture LD and biases ${\hat{N}}_{b}$ low (Neel et al. 2013; Whiteley et al. 2017). Similarly, incidental sampling above and below an instream barrier can induce the same phenomenon and bias ${\hat{N}}_{b}$ low.

There is little doubt that some the habitat patches sampled in this study are large enough that they do not represent a discrete population, such as the Savage River tributaries in Maryland, USA (Kazyak et al. 2016). However, in our data set, we did not observe a positive relationship between *F*_IS_ and habitat patch size that would be expected when pooling multiple genetic neighborhoods and we found a consistent positive relationship between patch size and ${\hat{N}}_{b}$. We would expect that at a certain habitat patch size, ${\hat{N}}_{b}$ would no longer increase proportionally with habitat size because the existence of more potential breeders does not apply at the scale of the *N*_b_ estimate. Our results suggest that being part of a larger habitat patch may have benefits that exceed the simple numerical addition of more breeders given more available habitat. Further work is needed to address sampling for and estimation of effective sizes in subdivided and continuously distributed populations and how such estimates should be interpreted within the context of existing conservation guidelines (Clarke et al. 2024; Ryman, Laikre, and Hössjer 2019).

## 5 Conclusions

Single‐sample estimates of effective number of breeders provide evidence for a link between a genetic metric and population status, temperature, base flow index, and available habitat at a basin wide scale in Brook Trout. Our results provide further support for the use of *N*_b_ for genetic monitoring, not only because it is sensitive to factors that affect Brook Trout population dynamics and occupancy but it is theoretically significant and summarizes factors that likely influence long‐term population viability, such as loss of genetic variation. In taxa with an identifiable age class, single‐sample estimates of *N*_b_ have the advantage of avoiding potential bias present in mixed‐age samples (Waples, Antao, and Luikart 2014), and can also be readily converted to generational effective size and placed into the context of existing international conservation frameworks (e.g., COP and CBD 2022). Additionally, single‐cohort genetic samples have other valuable applications such as identifying likely spawning locations using sibship reconstruction (Hudy et al. 2010) or assessing potential instream barriers (Whiteley et al. 2014a). Using the same single‐cohort samples, sibship‐derived summary statistics can be calculated, such as the number of families captured per unit sampling effort and variance in family size, which can provide closely related and ecologically meaningful information to an audience unfamiliar with population genetics. Importantly, expanding models such as the one we provide can aid in population prioritization and identify drivers of low *N*_b_ that are conducive to management action. The effective number of breeders and single‐cohort samples more generally provide powerful insights into a population and can likely complement many ongoing demographic and genetic monitoring programs in a diverse array of taxa.

## Conflicts of Interest

The authors declare no conflicts of interest.

## Supporting information


Appendices S1‐S3

## Data Availability

All genotypic data and environmental covariates used in this study are available at the Dryad Digital Repository: 10.5061/dryad.g4f4qrfzx.

## References

[eva13769-bib-0001] Ackerman, M. W. , B. K. Hand , R. K. Waples , et al. 2017. “Effective Number of Breeders From Sibship Reconstruction: Empirical Evaluations Using Hatchery Steelhead.” Evolutionary Applications 10, no. 2: 146–160. 10.1111/eva.12433. 28127391 PMC5253425

[eva13769-bib-0002] Aitken, K. E. H. , and K. Martin . 2012. “Experimental Test of Nest‐Site Limitation in Mature Mixed Forests of Central British Columbia, Canada.” Journal of Wildlife Management 76, no. 3: 557–565. 10.1002/jwmg.286.

[eva13769-bib-0003] Annett, B. , G. Gerlach , T. L. King , and A. R. Whiteley . 2012. “Conservation Genetics of Remnant Coastal Brook Trout Populations at the Southern Limit of Their Distribution: Population Structure and Effects of Stocking.” Transactions of the American Fisheries Society 141, no. 5: 1399–1410. 10.1080/00028487.2012.694831.

[eva13769-bib-0004] Bacles, C. F. E. , C. Bouchard , F. Lange , A. Manicki , C. Tentelier , and O. Lepais . 2018. “Estimating the Effective Number of Breeders From Single Parr Samples for Conservation Monitoring of Wild Populations of Atlantic Salmon *Salmo salar* .” Journal of Fish Biology 92, no. 3: 699–726. 10.1111/jfb.13537. 29377125

[eva13769-bib-0005] Bassar, R. D. , B. H. Letcher , K. H. Nislow , and A. R. Whiteley . 2016. “Changes in Seasonal Climate Outpace Compensatory Density‐Dependence in Eastern Brook Trout.” Global Change Biology 22, no. 2: 577–593. 10.1111/gcb.13135. 26490737

[eva13769-bib-0006] Beard, T. D. , and R. F. Carline . 1991. “Influence of Spawning and Other Stream Habitat Features on Spatial Variability of Wild Brown Trout.” Transactions of the American Fisheries Society 120, no. 6: 711–722. 10.1577/1548-8659(1991)120<0711:iosaos>2.3.co;2.

[eva13769-bib-0007] Bernos, T. A. , and D. J. Fraser . 2016. “Spatiotemporal Relationship Between Adult Census Size and Genetic Population Size Across a Wide Population Size Gradient.” Molecular Ecology 25, no. 18: 4472–4487. 10.1111/mec.13790. 27483203

[eva13769-bib-0008] Blum, A. G. , Y. Kanno , and B. H. Letcher . 2018. “Seasonal Streamflow Extremes Are Key Drivers of Brook Trout Young‐Of‐The‐Year Abundance.” Ecosphere 9, no. 8: e02356. 10.1002/ecs2.2356.

[eva13769-bib-0009] Brauer, C. J. , and L. B. Beheregaray . 2020. “Recent and Rapid Anthropogenic Habitat Fragmentation Increases Extinction Risk for Freshwater Biodiversity.” Evolutionary Applications 13, no. 10: 2857–2869. 10.1111/eva.13128. 33294027 PMC7691462

[eva13769-bib-0010] Campbell, N. R. , S. A. Harmon , and S. R. Narum . 2015. “Genotyping‐In‐Thousands by Sequencing (GT‐Seq): A Cost Effective SNP Genotyping Method Based on Custom Amplicon Sequencing.” Molecular Ecology Resources 15, no. 4: 855–867. 10.1111/1755-0998.12357. 25476721

[eva13769-bib-0011] Clarke, S. H. , E. R. Lawrence , J.‐M. Matte , et al. 2024. “Global Assessment of Effective Population Sizes: Consistent Taxonomic Differences in Meeting the 50/500 Rule.” Molecular Ecology 33: e17353. 10.1111/mec.17353. 38613250

[eva13769-bib-0012] Coombs, J. A. 2010. Reproduction in the Wild: The Effect of Individual Life History Strategies on Population Dynamics and Persistence. Amherst, Massachusetts: University of Massachusetts Amherst, *Dissertations* .

[eva13769-bib-0013] COP, & CBD . 2022. Kunming‐Montreal Global Biodiversity Framework. Montreal, Canada: UN Convention on Biological Diversity. https://www.cbd.int/doc/decisions/cop‐15/cop‐15‐dec‐04‐en.pdf.

[eva13769-bib-0014] Curry, R. A. , and D. L. G. Noakes . 1995. “Groundwater and the Selection of Spawning Sites by Brook Trout (*Salvelinus fontinalis*).” Canadian Journal of Fisheries and Aquatic Sciences 52, no. 8: 1733–1740. 10.1139/f95-765.

[eva13769-bib-0015] Curtis, S. M. “Mcmcplots: Create Plots From MCMC Output.” 2015. https://Cran.r‐Project.Org/Package=mcmcplots.

[eva13769-bib-0016] Delignette‐Muller, M. L. , and C. Dutang . 2015. “Fitdistrplus: An *R* Package for Fitting Distributions.” Journal of Statistical Software 64, no. 4: 1–34. 10.18637/jss.v064.i04.

[eva13769-bib-0017] DeWeber, J. T. , and T. Wagner . 2014. “Predicting Brook Trout Occurrence in Stream Reaches Throughout Their Native Range in the Eastern United States.” Transactions of the American Fisheries Society 144: 11–24. 10.1080/00028487.2014.963256.

[eva13769-bib-0018] Do, C. , R. S. Waples , D. Peel , G. M. Macbeth , B. J. Tillett , and J. R. Ovenden . 2014. “NeEstimator v2: Re‐Implementation of Software for the Estimation of Contemporary Effective Population Size (*N* _e_) From Genetic Data.” Molecular Ecology Resources 14, no. 1: 209–214. 10.1111/1755-0998.12157. 23992227

[eva13769-bib-0019] Dunham, J. , B. Rieman , and K. Davis . 2001. “Sources and Magnitude of Sampling Error in Redd Counts for Bull Trout.” North American Journal of Fisheries Management 21, no. 2: 343–352. 10.1577/1548-8675(2001)021<0343:samose>2.0.co;2.

[eva13769-bib-0020] Duong, T. Y. , K. T. Scribner , P. S. Forsythe , J. A. Crossman , and E. A. Baker . 2013. “Interannual Variation in Effective Number of Breeders and Estimation of Effective Population Size in Long‐Lived Iteroparous Lake Sturgeon (*Acipenser fulvescens*).” Molecular Ecology 22, no. 5: 1282–1294. 23293919 10.1111/mec.12167

[eva13769-bib-0021] EBTJV . 2016. “Range‐Wide Assessment of Brook Trout at the Catchment Scale: A Summary of Findings”.

[eva13769-bib-0022] Fesenmyer, K. A. , A. L. Haak , S. M. Rummel , M. Mayfield , S. L. McFall , and J. E. Williams . 2017. “Eastern Brook Trout Conservation Portfolio, Range‐Wide Habitat Integrity and Future Security Assessment, and Focal Area Risk and Opportunity Analysis ”.

[eva13769-bib-0023] Flanagan, S. P. , and A. G. Jones . 2019. “The Future of Parentage Analysis: From Microsatellites to SNPs and Beyond.” Molecular Ecology 28, no. 3: 544–567. 10.1111/mec.14988. 30575167

[eva13769-bib-0024] Franklin, I. R. 1980. “Evolutionary Change in Small Populations.” In Conservation Biology: An Evolutionary–Ecological Perspective, edited by M. E. Soule and B. A. Wilcox , 135–150. Sunderland, Massachusetts: Sinauer Associates.

[eva13769-bib-0025] Garner, B. A. , S. Hoban , and G. Luikart . 2020. “IUCN Red List and the Value of Integrating Genetics.” Conservation Genetics 21, no. 5: 795–801. 10.1007/s10592-020-01301-6.

[eva13769-bib-0026] Geist, D. R. , and D. D. Dauble . 1998. “Redd Site Selection and Spawning Habitat Use by Fall Chinook Salmon: The Importance of Geomorphic Features in Large Rivers.” Environmental Management 22, no. 5: 655–669. 9680535 10.1007/s002679900137

[eva13769-bib-0027] Gelman, A. 2013. “Two Simple Examples for Understanding Posterior p‐Values Whose Distributions Are Far From Uniform.” Electronic Journal of Statistics 7: 2595–2602. 10.1214/13-EJS854.

[eva13769-bib-0028] Gelman, A. , and D. B. Rubin . 1992. “Inference From Iterative Simulation Using Multiple Sequences.” Statistical Science 7, no. 4: 457–472.

[eva13769-bib-0029] Goudet, J. 2014. “Hierfstat: Estimation and Tests of Hierarchical F‐Statistics.” http://cran.r‐project.org/package=hierfstat.

[eva13769-bib-0030] Grant, G. C. , B. Vondracek , and P. W. Sorensen . 2002. “Spawning Interactions Between Sympatric Brown and Brook Trout May Contribute to Species Replacement.” Transactions of the American Fisheries Society 131, no. 3: 569–576. 10.1577/1548-8659(2002)131<0569:sibsba>2.0.co;2.

[eva13769-bib-0031] Hartman, K. J. , and J. P. Hakala . 2006. “Relationships Between Fine Sediment and Brook Trout Recruitment in Forested Headwater Streams.” Journal of Freshwater Ecology 21, no. 2: 215–230. 10.1080/02705060.2006.9664990.

[eva13769-bib-0032] Hitt, N. P. , E. L. Snook , and D. L. Massie . 2017. “Brook Trout Use of Thermal Refugia and Foraging Habitat Influenced by Brown Trout.” Canadian Journal of Fisheries and Aquatic Sciences 74, no. 3: 406–418. 10.1139/cjfas-2016-0255.

[eva13769-bib-0033] Hudy, M. , J. A. Coombs , K. H. Nislow , and B. H. Letcher . 2010. “Dispersal and Within‐Stream Spatial Population Structure of Brook Trout Revealed by Pedigree Reconstruction Analysis.” Transactions of the American Fisheries Society 139, no. 5: 1276–1287. 10.1577/T10-027.1.

[eva13769-bib-0034] Hudy, M. , D. M. Downey , and D. W. Bowman . 2000. “Successful Restoration of an Acidified Native Brook Trout Stream Through Mitigation With Limestone Sand.” North American Journal of Fisheries Management 20, no. 2: 453–466. 10.1577/1548-8675(2000)020<0453:SROAAN>2.3.CO;2.

[eva13769-bib-0035] Hudy, M. , T. M. Thieling , N. Gillespie , et al. 2008. “Distribution, Status, and Land Use Characteristics of Subwatersheds Within the Native Range of Brook Trout in the Eastern United States.” North American Journal of Fisheries Management 28, no. 4: 1069–1085. 10.1577/m07-017.1.

[eva13769-bib-0036] Huntsman, B. M. , J. T. Petty , S. Sharma , and E. R. Merriam . 2016. “More Than a Corridor: Use of a Main Stem Stream as Supplemental Foraging Habitat by a Brook Trout Metapopulation.” Oecologia 182, no. 2: 463–473. 10.1007/s00442-016-3676-4. 27334869

[eva13769-bib-0037] Jones, O. R. , and J. Wang . 2010. “COLONY: A Program for Parentage and Sibship Inference From Multilocus Genotype Data.” Molecular Ecology Resources 10, no. 3: 551–555. 10.1111/j.1755-0998.2009.02787.x. 21565056

[eva13769-bib-0038] Jorde, P. E. , and N. Ryman . 1995. “Temporal Allele Frequency Change and Estimation of Effective Size in Populations With Overlapping Generations.” Genetics 139, no. 2: 1077–1090. 7713410 10.1093/genetics/139.2.1077 PMC1206358

[eva13769-bib-0039] Kanno, Y. , M. A. Kulp , and S. E. Moore . 2016. “Recovery of Native Brook Trout Populations Following the Eradication of Nonnative Rainbow Trout in Southern Appalachian Mountains Streams.” North American Journal of Fisheries Management 36, no. 6: 1325–1335. 10.1080/02755947.2016.1221004.

[eva13769-bib-0040] Kanno, Y. , B. H. Letcher , N. P. Hitt , D. A. Boughton , J. E. B. Wofford , and E. F. Zipkin . 2015. “Seasonal Weather Patterns Drive Population Vital Rates and Persistence in a Stream Fish.” Global Change Biology 21, no. 5: 1856–1870. 10.1111/gcb.12837. 25523515

[eva13769-bib-0041] Kanno, Y. , K. C. Pregler , N. P. Hitt , B. H. Letcher , D. J. Hocking , and J. E. B. B. Wofford . 2016. “Seasonal Temperature and Precipitation Regulate Brook Trout Young‐Of‐The‐Year Abundance and Population Dynamics.” Freshwater Biology 61, no. 1: 88–99. 10.1111/fwb.12682.

[eva13769-bib-0042] Kanno, Y. , J. C. Vokoun , and B. H. Letcher . 2011. “Fine‐Scale Population Structure and Riverscape Genetics of Brook Trout (*Salvelinus fontinalis*) Distributed Continuously Along Headwater Channel Networks.” Molecular Ecology 20, no. 18: 3711–3729. 10.1111/j.1365-294X.2011.05210.x. 21819470

[eva13769-bib-0043] Kazyak, D. C. , R. H. Hilderbrand , T. L. King , S. R. Keller , and V. E. Chhatre . 2016. “Hiding in Plain Sight: A Case for Cryptic Metapopulations in Brook Trout (*Salvelinus fontinalis*).” PLoS One 11, no. 1: 1–18. 10.1371/journal.pone.0146295. PMC4701135 26730588

[eva13769-bib-0044] Kazyak, D. C. , B. A. Lubinski , M. A. Kulp , et al. 2022. “Population Genetics of Brook Trout in the Southern Appalachian Mountains.” Transactions of the American Fisheries Society 151, no. 2: 127–149. 10.1002/tafs.10337.

[eva13769-bib-0045] King, T. L. , M. S. Eackles , and B. H. Letcher . 2005. “Microsatellite DNA Markers for the Study of Atlantic Salmon (*Salmo salar*) Kinship, Population Structure, and Mixed‐Fishery Analyses.” Molecular Ecology Notes 5, no. 1: 130–132. 10.1111/j.1471-8286.2005.00860.x.

[eva13769-bib-0046] King, T. L. , B. A. Lubinski , M. K. Burnham‐Curtis , W. Stott , and R. P. Morgan II . 2012. “Tools for the Management and Conservation of Genetic Diversity in Brook Trout (*Salvelinus fontinalis*): Tri‐ and Tetranucleotide Microsatellite Markers for the Assessment of Genetic Diversity, Phylogeography, and Historical Demographics.” Conservation Genetics Resources 4, no. 3: 539–543. 10.1007/s12686-012-9603-z.

[eva13769-bib-0047] Koch, I. J. , and S. R. Narum . 2021. “An Evaluation of the Potential Factors Affecting Lifetime Reproductive Success in Salmonids.” Evolutionary Applications 14, no. 8: 1929–1957. 10.1111/eva.13263. 34429740 PMC8372082

[eva13769-bib-0048] Kuo, L. , and B. Mallick . 1998. “Variable Selection for Regression Models.” Sankhyā: The Indian Journal of Statistics, Series B (1960–2002) 60, no. 1: 65–81.

[eva13769-bib-0049] Laikre, L. 2020. “Post‐2020 Goals Overlook Genetic Diversity.” Science 367: 1083–1085. 10.1126/science.abb2748 32139534

[eva13769-bib-0050] Larson, G. L. , and S. E. Moore . 1985. “Encroachment of Exotic Rainbow Trout Into Stream Populations of Native Brook Trout in the Southern Appalachian Mountains.” Transactions of the American Fisheries Society 114, no. 2: 195–203. 10.1577/1548-8659(1985)114<195:EOERTI>2.0.CO;2.

[eva13769-bib-0051] Letcher, B. H. , K. H. Nislow , J. A. Coombs , M. J. O'Donnell , T. L. Dubreuil , and E. Svensson . 2007. “Population Response to Habitat Fragmentation in a Stream‐Dwelling Brook Trout Population.” PLoS One 2, no. 11: e1139. 10.1371/journal.pone.0001139. 18188404 PMC2190617

[eva13769-bib-0052] Letcher, B. H. , P. Schueller , R. D. Bassar , et al. 2014. “Robust Estimates of Environmental Effects on Population Vital Rates: An Integrated Capture–Recapture Model of Seasonal Brook Trout Growth, Survival and Movement in a Stream Network.” Journal of Animal Ecology 84, no. 2: 337–352. 10.1111/1365-2656.12308. 25327608

[eva13769-bib-0053] Linke, S. , E. Turak , and J. Nel . 2011. “Freshwater Conservation Planning: The Case for Systematic Approaches.” Freshwater Biology 56, no. 1: 6–20. 10.1111/j.1365-2427.2010.02456.x.

[eva13769-bib-0054] Luikart, G. , T. Antao , B. K. Hand , et al. 2021. “Detecting Population Declines via Monitoring the Effective Number of Breeders (*N* _b_).” Molecular Ecology Resources 21, no. 2: 379–393. 10.1111/1755-0998.13251. 32881365

[eva13769-bib-0055] Luikart, G. , N. Ryman , D. A. Tallmon , M. K. Schwartz , and F. W. Allendorf . 2010. “Estimation of Census and Effective Population Sizes: The Increasing Usefulness of DNA‐Based Approaches.” Conservation Genetics 11: 355–373. 10.1007/s10592-010-0050-7.

[eva13769-bib-0056] Makowski, D. , M. S. Ben‐Shachar , S. H. A. Chen , and D. Lüdecke . 2019. “Indices of Effect Existence and Significance in the Bayesian Framework.” Frontiers in Psychology 10. 10.3389/FPSYG.2019.02767/FULL. PMC6914840 31920819

[eva13769-bib-0057] Merriam, E. R. , J. T. Petty , and J. Clingerman . 2019. “Conservation Planning at the Intersection of Landscape and Climate Change: Brook Trout in the Chesapeake bay Watershed.” Ecosphere 10, no. 2: e02585. 10.1002/ecs2.2585.

[eva13769-bib-0058] Miller, L. M. , D. J. Dieterman , and R. J. H. Hoxmeier . 2019. “Reproductive Dynamics of a Native Brook Trout Population Following Removal of Non‐Native Brown Trout From a Stream in Minnesota, North‐Central USA.” Hydrobiologia 840, no. 1: 49–61. 10.1007/s10750-019-3952-5.

[eva13769-bib-0059] Mottl, O. , J. Yombai , T. M. Fayle , V. Novotný , and P. Klimeš . 2020. “Experiments With Artificial Nests Provide Evidence for Ant Community Stratification and Nest Site Limitation in a Tropical Forest.” Biotropica 52, no. 2: 277–287. 10.1111/btp.12684.

[eva13769-bib-0060] NADP . “National Atmospheric Deposition Program. Total Deposition Maps ” 2022. https://nadp.slh.wisc.edu/committees/tdep/.

[eva13769-bib-0061] Neel, M. C. , K. McKelvey , N. Ryman , et al. 2013. “Estimation of Effective Population Size in Continuously Distributed Populations: There Goes the Neighborhood.” Heredity 111, no. 3: 189–199. 10.1038/hdy.2013.37. 23652561 PMC3746818

[eva13769-bib-0062] Nislow, K. H. , and W. H. Lowe . 2003. “Influences of Logging History and Stream pH on Brook Trout Abundance in First‐Order Streams in New Hampshire.” Transactions of the American Fisheries Society 132, no. 1: 166–171. 10.1577/1548-8659(2003)132<0166:iolhas>2.0.co;2.

[eva13769-bib-0063] Nuhfer, A. J. , T. G. Zorn , and T. C. Wills . 2017. “Effects of Reduced Summer Flows on the Brook Trout Population and Temperatures of a Groundwater‐Influenced Stream.” Ecology of Freshwater Fish 26, no. 1: 108–119. 10.1111/eff.12259.

[eva13769-bib-0064] Petty, J. T. , P. J. Lamothe , and P. M. Mazik . 2005. “Spatial and Seasonal Dynamics of Brook Trout Populations Inhabiting a Central Appalachian Watershed.” Transactions of the American Fisheries Society 134, no. 3: 572–587. 10.1577/T03-229.1.

[eva13769-bib-0065] Plummer, M. 2003. “JAGS: A Program for Analysis of Bayesian Graphical Models Using Gibbs Sampling.” In Proceedings of the 3rd International Workshop on Distributed Statistical Computing (DSC 2003). 1:10. Vienna, Austria.

[eva13769-bib-0066] QGIS Development Team . “*QGIS* (3.18.3‐Zürich).” 2020. https://www.qgis.org.

[eva13769-bib-0067] R Core Team . “R: A Language and Environment for Statistical Computing.” 2020. https://www.r‐project.org/.

[eva13769-bib-0068] Rice, K. C. , and J. D. Jastram . 2015. “Rising Air and Stream‐Water Temperatures in Chesapeake Bay Region, USA.” Climatic Change 128, no. 1–2: 127–138. 10.1007/s10584-014-1295-9.

[eva13769-bib-0069] Robinson, Z. L. , J. A. Coombs , M. Hudy , K. H. Nislow , B. H. Letcher , and A. R. Whiteley . 2017. “Experimental Test of Genetic Rescue in Isolated Populations of Brook Trout.” Molecular Ecology 26, no. 17: 4418–4433. 10.1111/mec.14225. 28664980

[eva13769-bib-0070] Rouseset, F. 2008. “GENEPOP'007: A Complete Re‐Implementation of the GENEPOP Software for Windows and Linux.” Molecular Ecology 8, no. 1: 103–106. 10.1111/j.1471-8286.2007.01931.x. 21585727

[eva13769-bib-0071] Ruzzante, D. E. , G. R. McCracken , S. Parmelee , et al. 2016. “Effective Number of Breeders, Effective Population Size and Their Relationship With Census Size in an Iteroparous Species, *Salvelinus fontinalis* .” Proceedings of the Royal Society B: Biological Sciences 283, no. 1823. 10.1098/rspb.2015.2601. PMC4795031 26817773

[eva13769-bib-0072] Ryman, N. , L. Laikre , and O. Hössjer . 2019. “Do Estimates of Contemporary Effective Population Size Tell Us What We Want to Know?” Molecular Ecology 28, no. 8: 1904–1918. 10.1111/mec.15027. 30663828 PMC6850010

[eva13769-bib-0073] Saunders, J. W. , and M. W. Smith . 1965. “Changes in a Stream Population of Trout Associated With Increased Silt.” Journal of the Fisheries Research Board of Canada 22, no. 2: 395–404. 10.1139/f65-038.

[eva13769-bib-0074] Schofield, C. L. , and J. R. Trojnar . 1980. “Aluminum Toxicity to Brook Trout (*Salvelinus fontinalis*) in Acidified Waters.” In Polluted Rain, 341–366. New York: Springer US. 10.1007/978-1-4613-3060-8_18.

[eva13769-bib-0075] Su, Y. , and Y. Masanao . “*R2jags: Using R to Run “JAGS”* (R Package Version 0.5‐7).” 2015. https://cran.r‐project.org/package=R2jags.

[eva13769-bib-0076] Trumbo, B. A. , K. H. Nislow , J. Stallings , et al. 2014. “Ranking Site Vulnerability to Increasing Temperatures in Southern Appalachian Brook Trout Streams in Virginia: An Exposure‐Sensitivity Approach.” Transactions of the American Fisheries Society 143, no. 1: 173–187. 10.1080/00028487.2013.835282.

[eva13769-bib-0077] USGS . “National Hydrography Dataset Plus (2).” U.S. Dept. of the Interior, U.S. Geological Survey 2012. https://search.library.wisc.edu/catalog/9910061259502121.

[eva13769-bib-0078] Walker, J. D. , B. H. Letcher , and D. J. Hocking . “SHEDS Northeast Brook Trout Occupancy Model.” 2021. https://www.usgs.gov/apps/ecosheds/.

[eva13769-bib-0079] Wang, J. 2009. “A New Method for Estimating Effective Population Sizes From a Single Sample of Multilocus Genotypes.” Molecular Ecology 18, no. 10: 2148–2164. 10.1111/J.1365-294X.2009.04175.X. 19389175

[eva13769-bib-0080] Waples, R. S. 2002a. “Effective Size of Fluctuating Salmon Populations.” Genetics 161: 783–791. 12072473 10.1093/genetics/161.2.783 PMC1462121

[eva13769-bib-0081] Waples, R. S. 2002b. “Evaluating the Effect of Stage‐Specific Survivorship on the ne/N Ratio.” Molecular Ecology 11, no. 6: 1029–1037. 10.1046/J.1365-294X.2002.01504.X. 12090236

[eva13769-bib-0082] Waples, R. S. 2006. “A Bias Correction for Estimates of Effective Population Size Based on Linkage Disequilibrium at Unlinked Gene Loci.” Conservation Genetics 7, no. 2: 167–184. 10.1007/s10592-005-9100-y.

[eva13769-bib-0083] Waples, R. S. 2024. “Practical Application of the Linkage Disequilibrium Method for Estimating Contemporary Effective Population Size: A Review.” Molecular Ecology Resources 24, no. 1: e13879. 10.1111/1755-0998.13879. 37873672

[eva13769-bib-0084] Waples, R. S. , and T. Antao . 2014. “Intermittent Breeding and Constraints on Litter Size: Consequences for Effective Population Size per Generation (*N* _e_) and per Reproductive Cycle (*N* _b_).” Evolution 68, no. 6: 1722–1734. 10.1111/evo.12384. 24611912

[eva13769-bib-0085] Waples, R. S. , T. Antao , and G. Luikart . 2014. “Effects of Overlapping Generations on Linkage Disequilibrium Estimates of Effective Population Size.” Genetics 197, no. 2: 769–780. 10.1534/genetics.114.164822. 24717176 PMC4063931

[eva13769-bib-0086] Waples, R. S. , and C. Do . 2008. “LDNE: A Program for Estimating Effective Population Size From Data on Linkage Disequilibrium.” Molecular Ecology Resources 8, no. 4: 753–756. 10.1111/j.1755-0998.2007.02061.x. 21585883

[eva13769-bib-0087] Waples, R. S. , and C. Do . 2010. “Linkage Disequilibrium Estimates of Contemporary N_e_ Using Highly Variable Genetic Markers: A Largely Untapped Resource for Applied Conservation and Evolution.” Evolutionary Applications 3, no. 3: 244–262. 10.1111/j.1752-4571.2009.00104.x. 25567922 PMC3352464

[eva13769-bib-0088] Waples, R. S. , C. Do , and J. Chopelet . 2011. “Calculating *N* _e_ and *N* _e_/*N* in Age‐Structured Populations: A Hybrid Felsenstein‐Hill Approach.” Ecology 92: 1513–1522. 10.1890/10-1796.1. 21870625

[eva13769-bib-0089] Waples, R. S. , and P. R. England . 2011. “Estimating Contemporary Effective Population Size on the Basis of Linkage Disequilibrium in the Face of Migration.” Genetics 189, no. 2: 633–644. 10.1534/genetics.111.132233. 21840864 PMC3189803

[eva13769-bib-0090] Waples, R. S. , G. Luikart , J. R. Faulkner , and D. A. Tallmon . 2013. “Simple Life‐History Traits Explain Key Effective Population Size Ratios Across Diverse Taxa.” Proceedings of the Royal Society Series B 280: 20131339. 10.1098/rspb.2013.1339. PMC3757969 23926150

[eva13769-bib-0091] Waples, R. S. , and R. K. Waples . 2011. “Inbreeding Effective Population Size and Parentage Analysis Without Parents.” Molecular Ecology Resources 11, no. Suppl. 1: 162–171. 10.1111/j.1755-0998.2010.02942.x. 21429172

[eva13769-bib-0092] Waples, R. S. , and M. Yokota . 2007. “Temporal Estimates of Effective Population Size in Species With Overlapping Generations.” Genetics 175: 219–233. 10.1534/genetics.106.065300. 17110487 PMC1775005

[eva13769-bib-0093] Weber, N. , N. Bouwes , M. M. Pollock , et al. 2017. “Alteration of Stream Temperature by Natural and Artificial Beaver Dams.” PLoS One 12, no. 5: e0176313. 10.1371/journal.pone.0176313. 28520714 PMC5435143

[eva13769-bib-0094] Whiteley, A. R. , J. A. Coombs , M. Cembrola , et al. 2015. “Effective Number of Breeders Provides a Link Between Interannual Variation in Stream Flow and Individual Reproductive Contribution in a Stream Salmonid.” Molecular Ecology 24, no. 14: 3585–3602. 10.1111/mec.13273. 26080621

[eva13769-bib-0095] Whiteley, A. R. , J. A. Coombs , M. H. Hudy , Z. L. Robinson , K. H. Nislow , and B. H. Letcher . 2012. “Sampling Strategies for Estimating Brook Trout Effective Population Size.” Conservation Genetics 13, no. 3: 625–637. 10.1007/s10592-011-0313-y.

[eva13769-bib-0096] Whiteley, A. R. , J. A. Coombs , B. H. Letcher , and K. H. Nislow . 2014. “Simulation and Empirical Analysis of Novel Sibship‐Based Genetic Determination of Fish Passage.” Canadian Journal of Fisheries and Aquatic Sciences 71, no. 11: 1667–1679. 10.1139/cjfas-2014-0137.

[eva13769-bib-0097] Whiteley, A. R. , J. A. Coombs , M. J. O'Donnell , K. H. Nislow , and B. H. Letcher . 2017. “Keeping Things Local: Subpopulation *N* _b_ and *N* _e_ in a Stream Network With Partial Barriers to Fish Migration.” Evolutionary Applications 10, no. 4: 348–365. 10.1111/eva.12454. 28352295 PMC5367083

[eva13769-bib-0098] Whiteley, A. R. , M. Hudy , Z. L. Robinson , J. A. Coombs , and K. H. Nislow . 2014. “Patch‐Based Metrics: A Cost Effective Method for Short‐ and Long‐Term Monitoring of EBTJV Wild Brook Trout Populations?” In Proceedings of the Wild Trout XI Symposium, edited by R. F. Carline and C. LoSapio , 170–179. Bozeman, Montana: Wild Trout Symposium. https://www.wildtroutsymposium.com/proceedings.php.

[eva13769-bib-0104] Wieczorek, M. E. , S. E. Jackson , and G. E. Schwarz . 2018. "Select Attributes for NHDPlus Version 2.1 Reach Catchments and Modified Network Routed Upstream Watersheds for the Conterminous United States" (ver. 4.0, August 2023) [Data set]. U.S. Geological Survey. 10.5066/F7765D7V.

[eva13769-bib-0099] Wood, D. M. , A. B. Welsh , and J. Todd Petty . 2018. “Genetic Assignment of Brook Trout Reveals Rapid Success of Culvert Restoration in Headwater Streams.” North American Journal of Fisheries Management 38, no. 5: 991–1003. 10.1002/nafm.10185.

[eva13769-bib-0100] Wright, S. 1938. “Size of Population and Breeding Structure in Relation to Evolution.” Science 87: 430–431.

[eva13769-bib-0101] Wright, S. 1946. “Isolation by Distance Under Diverse Systems of Mating.” Genetics 31, no. 1: 39–59. 10.1093/genetics/31.1.39. 21009706 PMC1209315

[eva13769-bib-0102] Yates, M. C. , T. A. Bernos , and D. J. Fraser . 2017. “A Critical Assessment of Estimating Census Population Size From Genetic Population Size (Or Vice Versa) in Three Fishes.” Evolutionary Applications 10, no. 9: 935–945. 10.1111/eva.12496. 29151884 PMC5680432

[eva13769-bib-0103] Zhang, H. , T. Thieling , S. C. B. Prins , E. P. Smith , and M. Hudy . 2008. “Model‐Based Clustering in a Brook Trout Classification Study Within the Eastern United States.” Transactions of the American Fisheries Society 137, no. 3: 841–851.

