# Supplementary material for: Estimates of Effective Number of Breeders Identify Drivers of Decline in Mid‐Atlantic Brook Trout Populations
Source: Evol Appl. 2024 Sep 30;17(10):e13769. doi: 10.1111/eva.13769 (PMC11442137; doi:10.1111/eva.13769)
Supplement: Supplementary file 1 — Appendices S1‐S3 [file EVA-17-e13769-s001.docx]

**SUPPORTING INFORMATION**

# Estimates of effective number of breeders identify drivers of decline in mid-Atlantic Brook Trout populations.

**Appendix S1. Testing for Hardy-Weinberg proportions and linkage disequilibrium.**

We tested all populations genotyped using GT-seq for deviations from Hardy-Weinberg proportions (HWP) and for Linkage disequilibrium (LD) in the program GENEPOP version 4.7.5. Single cohort samples, such as the ones used in this study, have been shown to be out of global HWP and exhibit LD due to strong family structure in Brook Trout populations (Whiteley et al., 2013). As a result, we reject the null hypothesis these samples are in HWP or without linkage disequilibrium a priori, and instead use these tests to remove problematic loci. It’s worth highlighting, that we are using the LD signal of family structure and genetic drift to estimate *N*_b_, and with sufficient statistical power this will inevitably generate significant tests for LD. We filtered the marker panel using a binomial process approach for multiple testing across populations and markers similar to the approach demonstrated in Waples and Allendorf (2015). The goal of this testing is to remove loci that are likely physically linked, have null alleles present, or represent paralogous loci.

First, we performed tests for HW proportions for each of the 240 loci in *n* = 57 populations. In total, we conducted 9249 exact tests for HWP and 1034 (11%) were significant at $\alpha$ = 0.05. We filtered based on an expected probability of significant tests of $p$ = 0.25, rather than the traditional Type-1 error rate of 0.05 to account for elevated number of expected significant tests due to family structure. Assuming a locus can be tested in all populations (i.e., polymorphic in all populations), we expect on average to observe 14 significant tests ($n\times p$), and we would expect less than 5% of all loci to exhibit more than 20 significant tests. We removed all loci that had more significant tests more than the 5% threshold (20 significant tests) under the assumption of $p$ = 0.25. Note that we based the threshold of significant tests upon the actual number of tests conducted rather than maximum number of tests possible (i.e., 57).

Of the possible 1,634,760 LD tests assuming all loci were polymorphic, 917,633 LD tests were conducted. 160,264 (8.7%) of the tests were statistically significant at $\alpha$ = 0.05. We again filtered based on an expected probability of significant tests of $p$ = 0.25, which would correspond to an average of 17 expected significant tests per locus-pair comparison assuming the test was possible in each of the 57 populations. Under the expectation of $p$ = 0.25 for significant tests, less than 5% of locus-pair comparisons will exceed 20 significant tests out of 57 total tests. We iteratively dropped loci until all locus-pair comparisons had a proportion of significant tests no more than 20/57. In total, we removed 63 loci from consideration due to LD testing and 10 due to HWP testing. Following filtering based upon HWP and LD, we retained 167 SNP-loci for subsequent analysis.

**Appendix S2 Model file for** ${\hat{\boldsymbol{N}}}_{\mathbf{b}}$**, number full-sibling families (*N*_FAM_), and variance in family size (σ_FS_).** Note that observation weights are not applied for models of sibship-based summary statistics.

JAGS Model:

model {

##### Address covariates with missing data ########

for(j in 1:p){

for(i in 1:n){

X[i,j] ~ dnorm(muV[j],tauV[j])

}

tauV[j] ~ dunif(0,10)

muV[j] ~ dnorm(0,10e-20)

}

############################################

########### Random Intercept by state #########

sigma.state ~ dunif(0,10)

tau.state <- 1/(sigma.state*sigma.state)

muS ~ dnorm(0,10e-20)

for(i in 1:s){

ef[i] ~ dnorm(muS,tau.state)

}

for( i in 1:n){ # random effect

alpha[i]<- ef[state[i]]

}

#####################################

########### Coeff priors ###############

for (j in 1:p) {

beta[j] ~ dnorm(0,10e-20)

}

tau <- 1 / sigma * sigma

sigma ~ dunif(0,100)

####################################

######### Liklihood Statement ###############

for (i in 1:n) {

mu[i] <- alpha[i] +inprod(X[i,],beta)

y[i] ~ dlnorm(mu[i],tau/wt[i]) # note that weights are not applied to number full-sibling families (NFAM), and variance in family size (σFS).

}

##########################################

######### Evaluate Model Fit ##################

for(i in 1:n){

residual[i] <- log(y[i]) - mu[i]

predicted[i] <- mu[i]

sq[i] <- pow(residual[i],2)

# Generate replicate data

y.new[i] ~ dnorm(mu[i],tau/wt[i])

sq.new[i] <- pow(y.new[i] - predicted[i],2)

}

fit <- sum(sq[])

fit.new <- sum(sq.new[])

test <- step(fit.new - fit)

bpval <- mean(test) # Bayesian P-value

#############################################

}

**Appendix S3 Bayesian indicator model selection for models of *N*_b_**

Model and variable selection can aid in gauging the relative importance of each variable and suggest a more parsimonious model formulation. We provide an alternative model formulation for *N*_b_ estimates using Bayesian indicator variable selection *sensu* Kuo and Mallick (1998). The effects of each variable were estimated by sampling from the conditional spike and slab posterior $\theta_{j}=I_{j}\beta_{j}$ (O’Hara & Sillanpää, 2009). The probability of switching from $I_{j}$ = 0 to $I_{j}$ = 1 was estimated and was given an uninformative beta distributed prior ($\alpha$=1, $\beta$=1). Slope coefficients ($\beta_{j}$) of variables were estimated with uninformative, normally distributed priors centered on zero. However, to permit model convergence we allowed coefficients to share an estimated variance parameter. Similarly, we removed the random intercept for the U.S. state containing the habitat patch. We report the posterior probability of inclusion for each variable $\beta_{j}$ as the mean value of indicator $I_{j}$ (Table S1). Notably, the slope coefficients are consistent with the standard model in the main text, and are therefore redundant with straightforward interpretation of the posterior distributions (Figure S1).

**Table Appendix 3. Model results of Bayesian generalized linear mixed model.** Mean parameter estimates and 95% credible intervals are reported along with the proportion of iterations that a parameter was included in the model.

| Parameter | Mean  Estimate | 95% Credible Interval | *P*(*I*_j_=1) |
| --- | --- | --- | --- |
| Mean Intercept | 3.49 | 3.22 ­– 3.82 |  |
| Patch Size | 0.136 | -0.031 – 0.266 | 0.946 |
| Non-native salmonids | 0.057 | -0.224 – 0.290 | 0.702 |
| BFI | 0.152 | -0.019 – 0.294 | 0.953 |
| Mean Max Temperature | -0.204 | -0.332 – -0.080 | 0.996 |
| Deposition | 0.002 | -0.261 – 0.258 | 0.561 |
| Road Crossings | 0.138 | 0.050 – 0.215 | 0.993 |
| Canopy Cover | -0.053 | -0.233 – 0.221 | 0.706 |
| *pr*(*I*=1) | 0.715 | 0.369 – 0.955 |  |


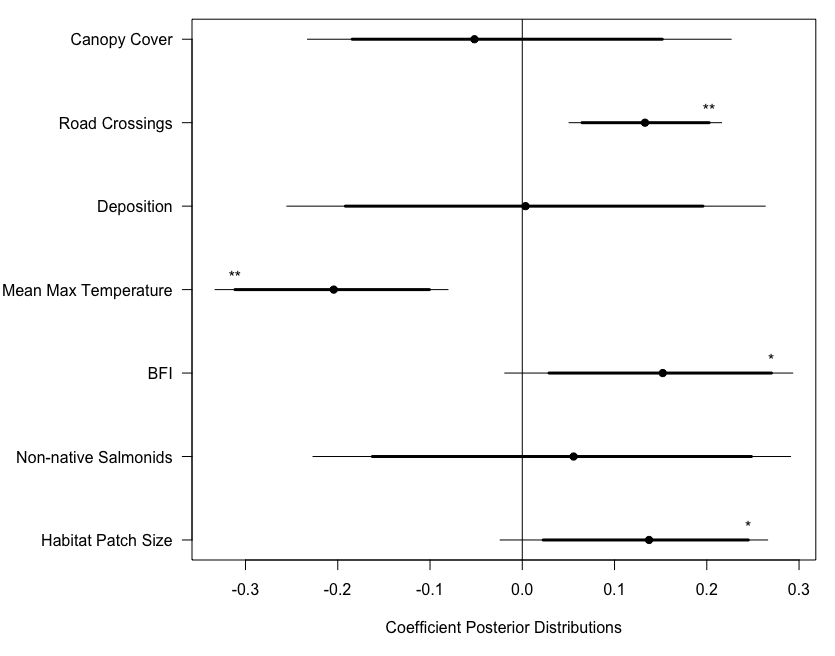


**Figure Appendix 3. Posterior distribution of slope coefficients for habitat patch size, non-native salmonid presence, BFI, mean maximum annual temperature, deposition, road crossings, and canopy cover when using indicator variable selection.** The mean estimate (point), 90% (darker line) and 95% (lighter line) credible intervals are reported. Statistical significance ($\alpha=0.05)$ is denoted with a one or two asterisks for one-tailed and two-tailed tests, respectively.

**Table S1. Description of variables included in models of *N*_b_.** The time period over which the variable applies, the hypothesized direction of effect on *N*_b_, and data source are also reported.

| Variable Name | Variable Time Period | Description | Hypothesis | Source |
| --- | --- | --- | --- | --- |
| Patch Size | – | Index of Available Habitat. Product of patch area (ha) and stream length (km). | Positive relationship | EBTJV and NHD+V2 |
| Presence of non-native salmonids | – | Index of competition with non-native salmonids | Negative relationship | EBTJV and Field Sampling Reports |
| Base Flow Index | 2003 | Index of flow and temperature stability and reproductive habitat. | Positive relationship | U.S. Geological Survey |
| Mean Max Air Temperature | 1991 – 2020 | Index of thermal environment | Negative relationship | PRISM Climate Group |
| Mean Cumulative Sulfur & Nitrogen Deposition | 2000-2002 2010-2020 | Index of acidifying precipitation | Negative relationship | National Atmospheric Deposition Program |
| Road Crossings per hectare | 2011 | Index of human activity and disturbance | Negative relationship | Delorme Road Layer |
| Percent Canopy Cover | 2016 | Index of anthropogenic land use and deforestation | Negative relationship | National Landcover Database |

**Table S2 Genetic summary statistics for each cohort sample.**

| PATCH_NAME | LAT | LONG | SAMPLE YEAR | MARKER  TYPE | Sample Size | *H*e | *F*_IS_ | ${\boldsymbol{LD}\hat{\boldsymbol{N}}}_{\boldsymbol{b}}$ | ${\boldsymbol{LD}\hat{\boldsymbol{N}}}_{\boldsymbol{b}}$-CI | *N*_FAM_ | μ_FAM_ | ${\boldsymbol{\sigma}^{\boldsymbol{2}}}_{\mathbf{FS}}$ | ${\boldsymbol{sib}\hat{\boldsymbol{N}}}_{\boldsymbol{b}}$ | ${\boldsymbol{sib}\hat{\boldsymbol{N}}}_{\boldsymbol{b}}\boldsymbol{CI}$ |
| --- | --- | --- | --- | --- | --- | --- | --- | --- | --- | --- | --- | --- | --- | --- |
| MD_BIGRUN | 39.54429 | -79.14102 | 2015 | MSAT | 75 | 0.781 | -0.030 | 285.4 | (134.1-2714.6) | 54 | 1.39 | 1.39 | 111 | (81-156) |
| MD_BIGRUN | 39.54429 | -79.14102 | 2017 | SNP | 43 | 0.176 | 0.025 | 1810.8 | (301.2-Inf) | 42 | 1.02 | 1.02 | 135 | (87-237) |
| MD_FISHINGCREEK | 39.52858 | -77.4713 | 2015 | MSAT | 75 | 0.766 | 0.037 | 146.2 | (88.2-305.5) | 52 | 1.44 | 1.44 | 107 | (76-154) |
| MD_FISHINGCREEK | 39.52858 | -77.4713 | 2017 | SNP | 59 | 0.188 | 0.034 | 179 | (113.7-355.2) | 45 | 1.31 | 1.31 | 104 | (72-161) |
| MD_MFCREEK | 39.51282 | -79.15446 | 2015 | MSAT | 68 | 0.778 | 0.042 | 140.8 | (89.8-263.8) | 54 | 1.26 | 1.26 | 84 | (58-124) |
| MD_MFCREEK | 39.51282 | -79.15446 | 2017 | SNP | 47 | 0.170 | 0.018 | 79.1 | (47.5-163.5) | 33 | 1.42 | 1.45 | 50 | (32-78) |
| MD_SAVAGE | 39.58545 | -79.09327 | 2015 | MSAT | 75 | 0.761 | -0.017 | 160.3 | (98.2-328.3) | 56 | 1.34 | 1.34 | 120 | (86-170) |
| MD_SAVAGE | 39.58545 | -79.09327 | 2017 | SNP | 50 | 0.176 | 0.051 | 126.1 | (69.7-353.6) | 43 | 1.16 | 1.16 | 142 | (92-254) |
| MD_WALKERSILVER | 39.68685 | -76.77257 | 2015 | MSAT | 75 | 0.681 | 0.060 | 72.4 | (45-128.7) | 42 | 1.79 | 1.91 | 55 | (38-80) |
| MD_WALKERSILVER | 39.68685 | -76.77257 | 2017 | SNP | 37 | 0.212 | -0.010 | 34.6 | (27.1-45.2) | 13 | 2.85 | 3.96 | 20 | (12-39) |
| NY_CARRS | 42.29251 | -75.23203 | 2015 | MSAT | 64 | 0.755 | 0.026 | 68 | (48.4-100.6) | 44 | 1.45 | 1.45 | 67 | (45-96) |
| NY_CARRS | 42.29251 | -75.23203 | 2016 | SNP | 76 | 0.311 | 0.046 | 47.6 | (35.6-64.9) | 33 | 2.30 | 4.38 | 30 | (19-50) |
| NY_CARRS | 42.29251 | -75.23203 | 2017 | SNP | 73 | 0.301 | 0.023 | 35.9 | (26.8-48.5) | 32 | 2.28 | 4.60 | 26 | (17-46) |
| NY_CHAMBERLAIN | 42.35152 | -77.42693 | 2015 | MSAT | 39 | 0.659 | -0.034 | 26.6 | (16.6-44.1) | 14 | 2.79 | 5.42 | 12 | (6-26) |
| NY_CHAMBERLAIN | 42.35152 | -77.42693 | 2016 | SNP | 49 | 0.285 | 0.029 | 49.7 | (34.4-76.1) | 29 | 1.69 | 1.69 | 42 | (26-65) |
| NY_CHAMBERLAIN | 42.35152 | -77.42693 | 2017 | SNP | 48 | 0.269 | 0.022 | 31.5 | (23.1-43.8) | 19 | 2.53 | 4.60 | 25 | (16-45) |
| NY_COHOCTON | 42.59696 | -77.53132 | 2015 | MSAT | 105 | 0.742 | 0.037 | 333.5 | (174.4-1251.9) | 81 | 1.30 | 1.30 | 123 | (89-167) |
| NY_COHOCTON | 42.59696 | -77.53132 | 2016 | SNP | 96 | 0.237 | 0.005 | 241.1 | (166-399.7) | 73 | 1.32 | 1.32 | 175 | (132-239) |
| NY_COHOCTON | 42.59696 | -77.53132 | 2017 | SNP | 34 | 0.234 | 0.007 | 187.3 | (99.8-790.1) | 29 | 1.17 | 1.17 | 99 | (62-185) |
| NY_FLYCREEK | 42.73794 | -74.97213 | 2015 | MSAT | 74 | 0.664 | 0.005 | 62.3 | (42.1-97.2) | 44 | 1.68 | 1.94 | 60 | (42-86) |
| NY_FLYCREEK | 42.73794 | -74.97213 | 2016 | SNP | 74 | 0.250 | 0.042 | 61 | (45.1-85) | 42 | 1.76 | 2.06 | 52 | (35-76) |
| NY_FLYCREEK | 42.73794 | -74.97213 | 2017 | SNP | 74 | 0.268 | 0.044 | 55.3 | (41.9-74.7) | 42 | 1.76 | 2.08 | 51 | (35-75) |
| NY_WILEYBROOK | 42.25988 | -75.6392 | 2016 | SNP | 17 | 0.261 | -0.006 | 40.2 | (25.9-75.3) | 14 | 1.21 | 1.21 | 75 | (38-310) |
| PA_BIGMILLCREEK | 41.5495 | -78.7903 | 2015 | MSAT | 151 | 0.786 | 0.012 | 117.2 | (85.7-165.3) | 95 | 1.59 | 1.90 | 101 | (77-138) |
| PA_BIRDRUN | 41.15586 | -77.56342 | 2016 | SNP | 109 | 0.217 | 0.022 | 97.8 | (72.8-136.2) | 62 | 1.76 | 1.98 | 89 | (64-125) |
| PA_EKAMMERDINER | 41.11929 | -77.33839 | 2016 | SNP | 86 | 0.177 | 0.022 | 68.4 | (53.4-89.3) | 45 | 1.91 | 2.28 | 57 | (39-83) |
| PA_ENGLISHRUN | 41.4428 | -77.29522 | 2018 | SNP | 78 | 0.225 | 0.017 | 123.2 | (87.1-189.1) | 54 | 1.44 | 1.44 | 112 | (84-153) |
| PA_LPLUMRUN | 41.2054 | -77.4074 | 2015 | MSAT | 72 | 0.632 | 0.134 | 27.2 | (16.1-45.1) | 31 | 2.32 | 3.51 | 32 | (20-52) |
| PA_MARTINRUN | 41.60392 | -79.07983 | 2016 | SNP | 35 | 0.184 | -0.034 | 15.3 | (10.3-21.9) | 8 | 4.38 | 11.80 | 13 | (7-30) |
| PA_MILLSTONE | 41.68228 | -76.51103 | 2015 | MSAT | 75 | 0.774 | 0.034 | 180.6 | (108.3-396.6) | 59 | 1.27 | 1.27 | 104 | (74-149) |
| PA_MILLSTONE | 41.68228 | -76.51103 | 2016 | SNP | 75 | 0.211 | 0.030 | 166.5 | (125.5-235.6) | 59 | 1.27 | 1.27 | 154 | (109-229) |
| PA_ROARINGRUN | 40.979 | -78.537 | 2015 | MSAT | 68 | 0.706 | 0.056 | 39.2 | (28.9-54.1) | 36 | 1.89 | 2.16 | 42 | (27-65) |
| PA_SBTIONESTACREEK | 41.62172 | -78.98412 | 2015 | MSAT | 45 | 0.716 | 0.040 | 175.3 | (85.7-1118.9) | 39 | 1.15 | 1.15 | 90 | (60-144) |
| PA_SBTIONESTACREEK | 41.62172 | -78.98412 | 2016 | SNP | 23 | 0.231 | 0.031 | 16.9 | (11.2-26.3) | 7 | 3.29 | 3.89 | 12 | (6-28) |
| PA_SEGLOCHRUN | 40.2324 | -76.27903 | 2015 | MSAT | 65 | 0.754 | -0.034 | 66.7 | (37.1-142.4) | 34 | 1.91 | 2.90 | 40 | (25-62) |
| PA_SHAEFFER | 40.26842 | -77.54943 | 2015 | MSAT | 75 | 0.725 | 0.031 | 275.2 | (138.2-1380.9) | 63 | 1.19 | 1.19 | 111 | (80-158) |
| PA_SHAEFFER | 40.26842 | -77.54943 | 2016 | SNP | 70 | 0.215 | 0.018 | 319.1 | (181.1-946.8) | 63 | 1.11 | 1.11 | 234 | (166-375) |
| PA_SIXMILERUN | 40.87103 | -78.11762 | 2018 | SNP | 71 | 0.280 | 0.019 | 64.5 | (45.6-95.7) | 43 | 1.65 | 1.97 | 58 | (40-85) |
| PA_WBTIONESTACREEK | 41.6964 | -79.0936 | 2015 | MSAT | 137 | 0.727 | 0.047 | 160.9 | (110-254.6) | 102 | 1.34 | 1.34 | 169 | (132-219) |
| VA_ABOVESWITZER | 38.5328 | -79.1747 | 2010 | MSAT | 379 | 0.780 | -0.041 | 66.6 | (59.4-74.5) | 116 | 3.27 | 11.18 | 95 | (72-128) |
| VA_ABOVESWITZER | 38.5328 | -79.1747 | 2011 | MSAT | 510 | 0.796 | -0.002 | 75.5 | (67.1-84.6) | 151 | 3.38 | 10.87 | 106 | (82-140) |
| VA_BARBOURSCREEK | 37.5599 | -80.0889 | 2013 | MSAT | 58 | 0.716 | 0.040 | 40.1 | (24.8-68.1) | 35 | 1.66 | 1.86 | 36 | (23-57) |
| VA_BLBRIERYBRANCH | 38.4382 | -79.1677 | 2011 | MSAT | 98 | 0.721 | 0.002 | 59.8 | (46.2-78.3) | 41 | 2.39 | 3.11 | 50 | (33-72) |
| VA_BLLITTLERIVER | 38.4111 | -79.162 | 2011 | MSAT | 57 | 0.645 | 0.020 | 69.8 | (40.4-143.9) | 27 | 2.11 | 3.20 | 32 | (21-52) |
| VA_BRIERYBRANCH | 38.4619 | -79.1848 | 2010 | MSAT | 72 | 0.728 | -0.007 | 26.2 | (18.7-36.4) | 27 | 2.67 | 6.55 | 29 | (18-50) |
| VA_BRIERYBRANCH | 38.4619 | -79.1848 | 2011 | MSAT | 90 | 0.701 | 0.049 | 32.4 | (24-43.7) | 34 | 2.65 | 7.31 | 19 | (11-38) |
| VA_BULLPASTURE | 38.34741 | -79.53484 | 2016 | SNP | 88 | 0.145 | 0.081 | 60.6 | (39-99.7) | 58 | 1.52 | 1.52 | 55 | (38-79) |
| VA_CUBRUN | 38.548911 | -78.642433 | 2016 | SNP | 77 | 0.234 | 0.080 | 55.9 | (41.9-76.4) | 47 | 1.64 | 2.06 | 41 | (27-65) |
| VA_CUBRUN | 38.548911 | -78.642433 | 2017 | SNP | 45 | 0.243 | 0.077 | 25.8 | (18.4-36.5) | 26 | 1.73 | 1.73 | 34 | (21-57) |
| VA_DRYRIVER | 38.5753 | -79.1027 | 2010 | MSAT | 143 | 0.778 | 0.002 | 335.9 | (218.2-617.3) | 107 | 1.34 | 1.34 | 150 | (117-194) |
| VA_DRYRIVER | 38.5753 | -79.1027 | 2011 | MSAT | 67 | 0.770 | 0.004 | 146.4 | (91.4-289) | 51 | 1.31 | 1.31 | 95 | (67-138) |
| VA_DRYRIVER | 38.5753 | -79.1027 | 2012 | MSAT | 86 | 0.761 | 0.002 | 125.1 | (78.5-232.1) | 57 | 1.51 | 1.72 | 62 | (43-89) |
| VA_DRYRUN | 38.55252 | -79.09682 | 2010 | MSAT | 46 | 0.565 | -0.051 | 4.9 | (3.1-11.3) | 20 | 2.30 | 3.00 | 21 | (12-41) |
| VA_DRYRUN | 38.55252 | -79.09682 | 2011 | MSAT | 27 | 0.392 | -0.117 | 40.2 | (13.1-Inf) | 18 | 1.50 | 1.50 | 93 | (57-182) |
| VA_EFELKCREEK | 37.57052 | -79.48894 | 2012 | MSAT | 74 | 0.514 | -0.041 | 16.7 | (8.7-27.9) | 26 | 2.84 | 8.80 | 16 | (9-34) |
| VA_EFELKCREEK | 37.57052 | -79.48894 | 2018 | SNP | 98 | 0.140 | 0.034 | 48.1 | (37.2-62.6) | 32 | 3.06 | 6.93 | 34 | (22-53) |
| VA_ENCHANTEDCREEK | 37.66056 | -79.29326 | 2018 | SNP | 79 | 0.145 | 0.010 | 45 | (34.4-59.4) | 30 | 2.63 | 4.92 | 40 | (26-62) |
| VA_FRIDLEY | 38.49751 | -78.7149 | 2009 | MSAT | 69 | 0.773 | 0.076 | 82.5 | (55.2-134.5) | 44 | 1.57 | 1.57 | 63 | (43-94) |
| VA_FRIDLEY | 38.49751 | -78.7149 | 2010 | MSAT | 99 | 0.788 | 0.051 | 128.9 | (80.9-236.6) | 61 | 1.62 | 1.62 | 78 | (57-111) |
| VA_FRIDLEY | 38.49751 | -78.7149 | 2011 | MSAT | 211 | 0.775 | 0.055 | 104 | (86-126.4) | 102 | 2.07 | 2.87 | 101 | (77-137) |
| VA_FRIDLEY | 38.49751 | -78.7149 | 2018 | SNP | 75 | 0.179 | 0.005 | 62.4 | (48.2-82.8) | 35 | 2.14 | 3.31 | 46 | (31-70) |
| VA_GUYSRUN | 37.92789 | -79.49866 | 2012 | MSAT | 76 | 0.542 | 0.017 | 43.8 | (27.2-73.1) | 48 | 1.58 | 2.03 | 34 | (21-52) |
| VA_GUYSRUN | 37.92789 | -79.49866 | 2015 | MSAT | 50 | 0.565 | 0.055 | 42.8 | (23.5-89) | 30 | 1.67 | 1.76 | 35 | (22-56) |
| VA_JORDANRUN | 38.0503 | -79.72272 | 2015 | MSAT | 24 | 0.606 | -0.062 | 26.8 | (14-64.1) | 15 | 1.60 | 1.60 | 16 | (8-38) |
| VA_LITTLEBACKCR | 38.17128 | -79.88097 | 2015 | MSAT | 46 | 0.613 | 0.001 | 65.9 | (38.6-136.9) | 34 | 1.35 | 1.35 | 54 | (34-84) |
| VA_LITTLEIRISH | 37.67759 | -79.29237 | 2018 | SNP | 92 | 0.185 | 0.003 | 37.9 | (30.4-47.3) | 28 | 3.29 | 8.72 | 28 | (18-47) |
| VA_LITTLERIVER | 38.4087 | -79.1936 | 2010 | MSAT | 299 | 0.712 | 0.013 | 46 | (37.8-55.4) | 112 | 2.67 | 8.08 | 63 | (45-89) |
| VA_LITTLERIVER | 38.4087 | -79.1936 | 2011 | MSAT | 383 | 0.717 | 0.026 | 54.7 | (46-64.6) | 117 | 3.27 | 8.52 | 96 | (73-129) |
| VA_LITTLERIVER | 38.4087 | -79.1936 | 2015 | MSAT | 28 | 0.738 | -0.128 | 29 | (13.3-89.3) | 15 | 1.87 | 3.98 | 17 | (9-36) |
| VA_LITTLESTONYNEW | 37.3999 | -80.5161 | 2013 | MSAT | 43 | 0.601 | -0.019 | 44.8 | (19.5-152.1) | 27 | 1.59 | 1.59 | 39 | (24-63) |
| VA_LITTLESTONYSHEN | 38.9492 | -78.6365 | 2012 | MSAT | 75 | 0.727 | -0.005 | 130.9 | (80.5-258.1) | 50 | 1.50 | 1.50 | 74 | (53-106) |
| VA_LITTLEWILSON | 37.9893 | -79.7699 | 2012 | MSAT | 58 | 0.556 | 0.034 | 11.6 | (6-17.7) | 17 | 3.41 | 10.34 | 15 | (9-31) |
| VA_LPASSAGECREEK | 38.91795 | -78.37998 | 2017 | SNP | 24 | 0.194 | 0.041 | 19.8 | (12.8-32.3) | 11 | 2.18 | 4.11 | 12 | (6-26) |
| VA_MILLRUN | 38.87663 | -78.36936 | 2017 | SNP | 27 | 0.215 | -0.013 | 10.5 | (4.6-18.9) | 4 | 6.75 | 64.78 | 4 | (2-15) |
| VA_NFBUFFALORIVER | 37.7084 | -79.2039 | 2012 | MSAT | 75 | 0.654 | -0.027 | 62.7 | (39.6-106.9) | 43 | 1.74 | 2.09 | 58 | (40-83) |
| VA_NFTYERIVER | 37.88139 | -79.11146 | 2012 | MSAT | 73 | 0.716 | -0.040 | 231.1 | (119.5-906.3) | 55 | 1.33 | 1.33 | 87 | (62-125) |
| VA_NFTYERIVER | 37.88139 | -79.11146 | 2015 | MSAT | 75 | 0.692 | -0.003 | 227.2 | (71.9-Inf) | 61 | 1.23 | 1.23 | 103 | (77-146) |
| VA_NOBUSINESS | 37.248 | -80.8922 | 2013 | MSAT | 52 | 0.739 | 0.071 | 86.4 | (53.6-165.7) | 33 | 1.58 | 1.58 | 62 | (41-94) |
| VA_NORTHRIVER | 38.37348 | -79.27921 | 2015 | MSAT | 104 | 0.744 | 0.020 | 129.4 | (66-352) | 70 | 1.49 | 1.49 | 103 | (75-145) |
| VA_NORTHRIVER | 38.37348 | -79.27921 | 2016 | SNP | 42 | 0.167 | 0.037 | 120 | (67.5-329) | 37 | 1.35 | 1.35 | 105 | (70-187) |
| VA_PEARISTHOMPSON | 37.2107 | -80.9053 | 2013 | MSAT | 18 | 0.376 | 0.105 | 6.9 | (2.2-27.5) | 13 | 1.38 | 1.38 | 16 | (8-43) |
| VA_PEDLARRIVER | 37.7549 | -79.2685 | 2012 | MSAT | 59 | 0.735 | -0.015 | 26.8 | (19.3-37.3) | 23 | 2.57 | 5.80 | 22 | (13-42) |
| VA_RAMSEYDRAFT | 38.34055 | -79.34476 | 2015 | MSAT | 58 | 0.625 | 0.012 | -2776.2 | (209.5-Inf) | 47 | 1.23 | 1.23 | 100 | (70-148) |
| VA_RAMSEYDRAFT | 38.34055 | -79.34476 | 2016 | SNP | 50 | 0.174 | 0.056 | 138.9 | (89.1-266.9) | 37 | 1.35 | 1.35 | 87 | (56-147) |
| VA_ROBERTSCREEK | 37.71387 | -79.2903 | 2018 | SNP | 27 | 0.184 | -0.041 | 15 | (9.5-24) | 4 | 6.75 | 28.74 | 6 | (3-21) |
| VA_SFNORTHRIVER | 38.37362 | -79.22571 | 2010 | MSAT | 50 | 0.542 | 0.120 | 10.1 | (1.4-60.9) | 24 | 2.08 | 3.51 | 16 | (9-34) |
| VA_SFNORTHRIVER | 38.37362 | -79.22571 | 2011 | MSAT | 50 | 0.519 | -0.004 | 17.1 | (10.9-25.8) | 18 | 2.78 | 4.89 | 15 | (8-31) |
| VA_SOBUFFALOCREEK | 37.723 | -79.592 | 2012 | MSAT | 75 | 0.547 | -0.034 | 29.8 | (20.4-43.2) | 33 | 2.27 | 4.00 | 29 | (19-50) |
| VA_STANDROCKBRANCH | 37.21412 | -80.8929 | 2013 | MSAT | 60 | 0.531 | -0.018 | 20.5 | (10.9-36.3) | 33 | 1.82 | 2.46 | 28 | (17-48) |
| VA_STONYCREEKNEW | 37.4372 | -80.5396 | 2013 | MSAT | 52 | 0.755 | 0.128 | 24.6 | (16.2-37.4) | 26 | 2.00 | 2.00 | 37 | (24-61) |
| VA_UNIONSPRINGS | 38.47386 | -79.0806 | 2018 | SNP | 50 | 0.182 | 0.071 | 70.2 | (49.2-108.7) | 32 | 1.56 | 1.56 | 51 | (32-78) |
| VA_WILSONCREEK | 37.93864 | -79.80486 | 2012 | MSAT | 65 | 0.522 | -0.075 | 67.7 | (37.9-143.8) | 38 | 1.71 | 1.71 | 73 | (52-103) |
| VA_WILSONCREEK | 37.93864 | -79.80486 | 2015 | MSAT | 25 | 0.538 | 0.033 | 99.5 | (29.4-Inf) | 19 | 1.32 | 1.32 | 53 | (28-164) |
| VA_WILSONCREEK | 37.93864 | -79.80486 | 2016 | SNP | 41 | 0.118 | 0.032 | 38 | (23-69.2) | 26 | 1.58 | 1.58 | 41 | (27-67) |
| WV_BRIGGSRUN | 38.822 | -79.295 | 2017 | SNP | 31 | 0.141 | 0.037 | 17.9 | (11.9-27.7) | 16 | 1.94 | 2.24 | 18 | (10-38) |
| WV_CLUBHOUSERUN | 38.63358 | -79.75668 | 2018 | SNP | 59 | 0.064 | 0.053 | 24.8 | (3.9-156) | 35 | 1.69 | 1.69 | 44 | (28-69) |
| WV_DEERRUN | 38.7711 | -79.2613 | 2017 | SNP | 35 | 0.162 | 0.021 | 39 | (25.3-65.7) | 17 | 2.06 | 4.17 | 13 | (7-30) |
| WV_FIVEMILEHOLLOW | 38.58452 | -79.71896 | 2018 | SNP | 68 | 0.129 | 0.039 | 85.8 | (57.1-142.7) | 43 | 1.58 | 1.58 | 68 | (47-99) |
| WV_HAWKRUN | 39.135443 | -78.532192 | 2017 | SNP | 35 | 0.201 | 0.034 | 34 | (23.3-52.1) | 18 | 1.94 | 2.68 | 23 | (14-42) |
| WV_HIMMELWRIGHTRUN | 39.138878 | -78.475051 | 2017 | SNP | 45 | 0.136 | 0.018 | 39.7 | (23.8-73.4) | 27 | 1.67 | 1.94 | 40 | (24-67) |
| WV_LUNICECREEK | 39.1564 | -79.2164 | 2017 | SNP | 34 | 0.097 | -0.003 | 27.6 | (16.6-49.3) | 18 | 1.89 | 2.62 | 21 | (12-41) |
| WV_MILLRUNKLINE | 38.778 | -79.225 | 2017 | SNP | 32 | 0.180 | 0.017 | 80.4 | (43.7-240.7) | 29 | 1.10 | 1.10 | 142 | (81-368) |
| WV_NORTHRIVER | 39.126782 | -78.821261 | 2017 | SNP | 36 | 0.235 | -0.014 | 20.1 | (14.4-28.4) | 13 | 2.77 | 4.65 | 19 | (11-38) |
| WV_REEDSCREEK | 38.76442 | -79.32007 | 2018 | SNP | 83 | 0.197 | 0.025 | 123.5 | (92.6-173.6) | 53 | 1.57 | 1.57 | 96 | (68-141) |
| WV_SPANOAKRUN | 38.62495 | -79.77852 | 2018 | SNP | 78 | 0.109 | 0.079 | 21.3 | (14.1-31.2) | 46 | 1.70 | 1.75 | 41 | (27-65) |
| WV_TROUTFRANKLIN | 38.674 | -79.298 | 2017 | SNP | 49 | 0.160 | 0.027 | 119.4 | (54.9-765.5) | 41 | 1.20 | 1.20 | 120 | (82-194) |

**Table S3. Table of Pearson’s Correlation values among variables used in models of** ${\hat{\boldsymbol{N}}}_{\mathbf{b}}$**, number full-sibling families (*N*_FAM_), and variance in family size (σ_FS_).**

|  | Patch Size | Non-native trout | Base flow index | Mean Max Temp. | Deposition | Road Crossings per ha | Percent Canopy Cover |
| --- | --- | --- | --- | --- | --- | --- | --- |
| Patch Size | 1.000 |  |  |  |  |  |  |
| Non-native trout | 0.277 | 1.000 |  |  |  |  |  |
| Base flow index | -0.120 | 0.032 | 1.000 |  |  |  |  |
| Mean Max Temp. | -0.051 | -0.413 | 0.305 | 1.000 |  |  |  |
| Acid Deposition | -0.079 | 0.339 | 0.490 | -0.229 | 1.000 |  |  |
| Road Crossings per ha | -0.083 | 0.071 | 0.117 | 0.027 | -0.064 | 1.000 |  |
| Percent Canopy Cover | -0.093 | -0.219 | 0.044 | 0.434 | -0.070 | -0.226 | 1.000 |

**Figure S1. Comparison of effective number of breeder estimates based on the GT-seq marker panel (SNP), and those based on microsatellites (MSAT) for North River, Virginia Brook Trout habitat patch**. Two different critical allele frequencies (0.02 and 0.05) were used within the program NeEstimator V2.1.


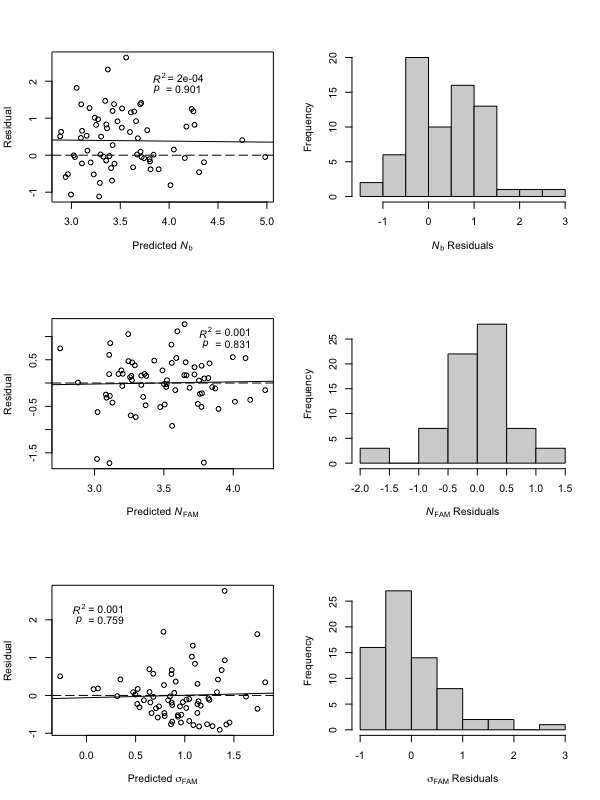


**Figure S2.** **Relationship between residuals and predicted values in models of** ${\hat{\boldsymbol{N}}}_{\mathbf{b}}$**, number full-sibling families (*N*_FAM_), and variance in family size (σ^2^_FS_).** The *p*-values and *R*^2^ from simple linear regression are reported for residual and predicted value relationships.
